# Reactive sulfur species and their significance in health and disease

**DOI:** 10.1042/BSR20221006

**Published:** 2022-09-14

**Authors:** Małgorzata Iciek, Anna Bilska-Wilkosz, Michał Kozdrowicki, Magdalena Górny

**Affiliations:** Chair of Medical Biochemistry, Faculty of Medicine, Jagiellonian University Medical College, Kopernika 7, 31-034 Krakow, Poland

**Keywords:** hydrogen sulfide, persulfides, polysulfides, protein persulfidation, reactive sulfur species

## Abstract

Reactive sulfur species (RSS) have been recognized in the last two decades as very important molecules in redox regulation. They are involved in metabolic processes and, in this way, they are responsible for maintenance of health. This review summarizes current information about the essential biological RSS, including H_2_S, low molecular weight persulfides, protein persulfides as well as organic and inorganic polysulfides, their synthesis, catabolism and chemical reactivity. Moreover, the role of RSS disturbances in various pathologies including vascular diseases, chronic kidney diseases, diabetes mellitus Type 2, neurological diseases, obesity, chronic obstructive pulmonary disease and in the most current problem of COVID-19 is presented. The significance of RSS in aging is also mentioned. Finally, the possibilities of using the precursors of various forms of RSS for therapeutic purposes are discussed.

## Introduction

**Figure 1:**
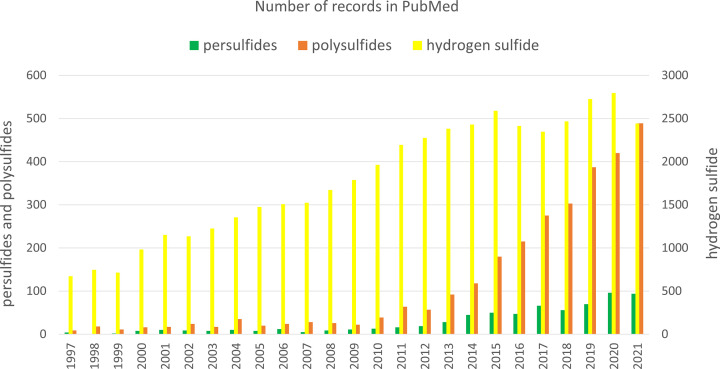
The number of records in PubMed obtained by searching the database for RSS that included H_2_S (yellow), persulfides (green) and polysulfides (brown) published over the last 25 years

Sulfur is an essential element for living organisms and one of the most abundant elements on earth. Plants and numerous microorganisms absorb sulfur from the soil in the form of sulfate (SO_4_^2−^), which is then reduced to S^2−^ and incorporated into sulfur amino acids, methionine and cysteine [1]. In animals, sulfur is absorbed from the digestive tract in the form of sulfur amino acids, of which methionine is an essential amino acid, while cysteine can be produced endogenously from methionine; therefore, it is not an essential amino acid. Reactive sulfur species (RSS), including hydrogen sulfide (H_2_S), persulfides and polysulfides are synthetized in all living organisms mainly from cysteine and have been recognized in the last two decades as very important molecules in redox regulation. They participate in sulfur trafficking and are involved in metabolic processes and in this way, they are responsible for maintenance of health. The important role of RSS in the regulation of biological processes is reflected by the expanding interest of scientists and the ever-growing number of studies on individual biologically active forms of sulfur. Figure 1 shows the number of records in PubMed for the search terms: H_2_S, persulfides and polysulfides published over the last 25 years. Since 1997, there has been a steady increase in interest in all these RSS forms that proves that this topic is up-to-date and offers opportunities for new discoveries.

There are many evidence that the disruption of RSS homeostasis leads to some pathologies. At the same time, it was also shown that the modulation of RSS levels in the cell by using their precursors could be a potential and promising therapeutic tool. Recently, the significance of RSS has also been investigated in relation to the infection caused by SARS-CoV-2 virus, and some potential donors or activators of endogenous RSS synthesis are tested as a preventive treatment in COVID-19.

## H_2_S and its properties

Hydrogen sulfide (H_2_S) is probably the best known form of RSS. The story of H_2_S as a physiological regulator started in the 1990s, when Kimura et al. documented that this small gaseous molecule was produced endogenously in the nervous system, where it fulfilled an important role [2]. After that many other studies have found that H_2_S is synthesized also in other animal tissues and its physiological role has been widely discussed [3–5]. This interest in the biological significance of H_2_S and research on its endogenous synthesis and catabolism was reflected in a large increase in the number of publications regarding this topic (Figure 1).

However, the beginning of discoveries related to endogenous H_2_S dates back 50 years earlier, when the transsulfuration pathway, involving the interconversion of cysteine and homocysteine, through the intermediate cystathionine, was discovered in animal liver and other tissues. During that study, the authors described the production of H_2_S, but they did not recognize the biological potential and significance of this gaseous mediator [6].

H_2_S is the one of the three well-known gasotransmitters. The feature that distinguishes it from other gasotransmitters (carbon monoxide, CO and nitric oxide, NO) is its characteristic odor and ability to dissociate. As a weak acid, H_2_S dissociates in two stages to hydrosulfide anion (HS^−^) and sulfide anion (S^2−^) (reaction 1):

(1) $${\text{H}}_{2}\text{S}⇄{\text{HS}}^{-}⇄{\text{S}}^{2-}$$

Deprotonation of H_2_S to form HS^−^ has a p*K*_a_≈7.0, while the second p*K*_a_ value is 13.1. It means that under physiological pH in extracellular fluids (e.g. plasma), H_2_S exists predominantly as HS^−^ (80%), concentration of S^2−^ is negligible while H_2_S accounts for nearly 20%. Inside the cell, in the cytosol, where pH value is a little lower (≈7.0), approximately 50% of H_2_S exists as HS^−^ anion. The proportion of the different ionized forms of H_2_S may vary depending on the prevailing pH in the specific cell organelles. In mitochondria, where the pH is above 7.8, the concentration of the HS^−^ has been estimated to represent approximately 92%. On the other hand, in lysosomes, where the environment is acidic (pH 4.7), nearly 99% of H_2_S is present in the undissociated form and the HS^−^ anion content is below 1% [7]. H_2_S, as a more hydrophobic molecule, freely penetrates the lipid bilayer of the cell membrane by simple diffusion. HS^−^ anion is more hydrophilic, so it is transported by facilitated diffusion and requires special transporters, such as anion exchange protein AE1 present in erythrocytes [8]. It is assumed that the term hydrogen sulfide (H_2_S) is used to describe all forms of H_2_S existing in a pH-dependent equilibrium in the cellular environment. In this review, similarly, the term covers all forms of H_2_S.

### Sources of endogenous H_2_S

**Figure 2:**
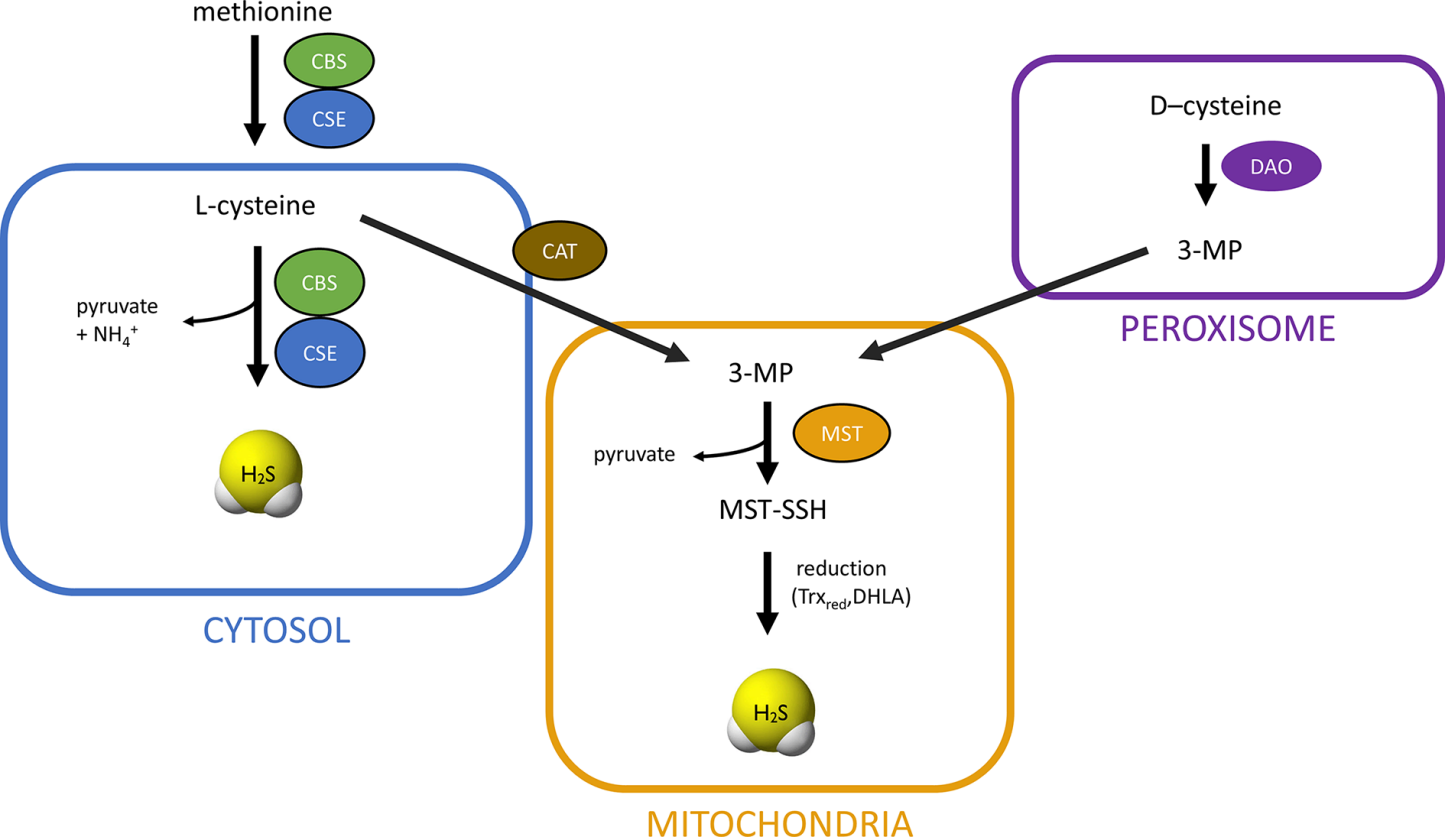
Biosynthetic pathway leading to H_2_S production from L- and D-cysteine L-cysteine is synthesized endogenously from methionine. It is converted to L-homocysteine that reacts with L-serine forming cystathionine in the reaction catalyzed by CBS. Cystathionine is then cleaved by CSE to L-cysteine and L-homoserine. L-cysteine is a substrate for reactions leading to H_2_S generation presented in Figure 3. L-cysteine can be also a substrate for CAT, which results in the formation of 3-MP. The latter compound is a substrate for MST that temporarily creates a persulfide form and then H_2_S is released under the influence of reducing agents: Trx_red_ or DHLA. The pathway of H_2_S synthesis from D-cysteine exists mainly in peroxisomes, where DAO converts it to 3-MP.

**Figure 3:**
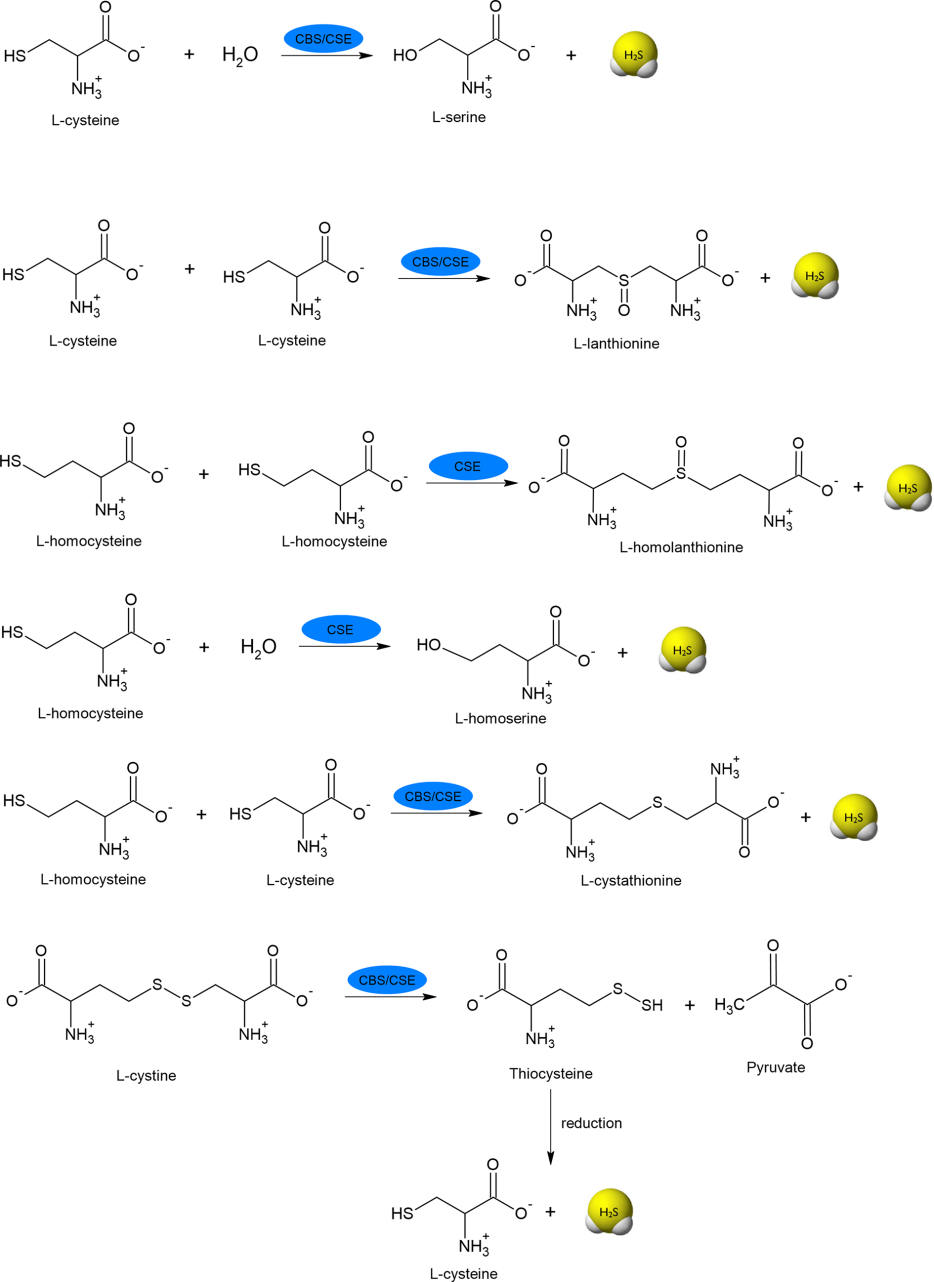
Specific reactions catalyzed by CBS and CSE responsible for H_2_S generation

H_2_S is produced endogenously in mammalian cells mainly from L-cysteine (Cys-SH) and this process is catalyzed by three enzymes: cystathionine β-synthase (CBS), cystathionine γ-lyase (also known as cystathionase, CSE) and 3-mercaptopyruvate sulfurtransferase (MST). CBS and CSE are pyridoxal phosphate (PLP) dependent and are localized in the cytosol whereas MST is PLP independent and resides in mitochondria. CBS and CSE participate in the transsulfuration pathway that converts L-homocysteine (derived from methionine) into Cys-SH (Figure 2). Moreover, these two enzymes are responsible for H_2_S generation in many additional reactions (Figure 3).

Cys-SH can be transformed to 3-mercaptopyruvate (3-MP) in reaction catalyzed by PLP-dependent cysteine aminotransferase (CAT). The product of this reaction, 3-MP, is a substrate for MST, which takes on a sulfur atom forming intermediate MST-SSH and then H_2_S is released under the influence of reducing agents, e.g. thioredoxin (Trx_red_) or dihydrolipoic acid (DHLA) (Figure 2) [9,10].

**Figure 4:**
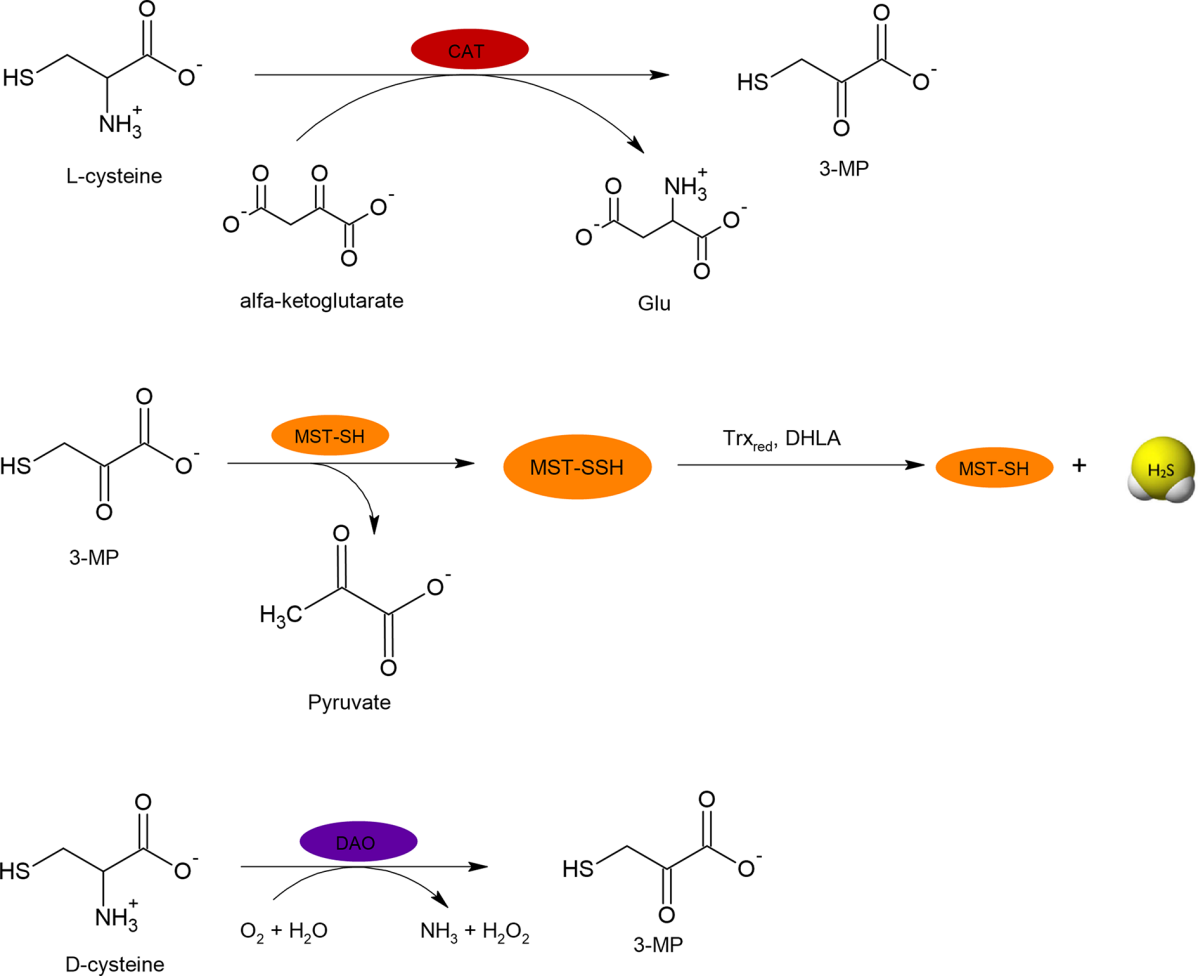
Reactions catalyzed by CAT, MST and DAO responsible for H_2_S generation

In peroxisomes, there is another possibility of H_2_S synthesis, namely from D-cysteine. In the first step, FMN-dependent D-amino acid oxidase (DAO), occurring in these organelles, converts D-cysteine to 3-MP, which then is a substrate for the MST [11]. The reactions catalyzed by CAT, MST and DAO are presented in Figure 4. Moreover, information about enzymes involved in H_2_S synthesis and their substrates are summarized in Table 1.

**Table 1:** Enzymes involved in synthesis of various RSS

Enzyme involved in RSS synthesis	Kind of RSS formed by the enzyme	Substrate for RSS synthesis
CBS^*^	H_2_S, persulfides	L-cysteine, L-homocysteine, L-cystine
CSE*	H_2_S, persulfides	L-cysteine, L-homocysteine, L-cystine
MST	polysulfides	3-MP
CAT	H_2_S	L-cysteine
DAO	H_2_S	D-cysteine
SQR	persulfides	GSH, CoA-SH
GR	persulfides	GSSSG
CARS	persulfides, polysulfides	L-cysteine

* Pirydoxal phosphate dependent enzyme.

The main enzymes involved in endogenous H_2_S synthesis (CBS, CSE and MST) are expressed in many tissues. The highest expression of all three enzymes has been documented in the liver. CBS is mainly expressed in the brain, liver, kidney and pancreas. CSE has been found predominantly in the liver, kidney, thoracic aorta, ileum, portal vein, uterus, pancreatic islets and in the placenta. A modest expression and activity of CSE has been shown in the brain. MST, apart from the liver, is expressed also in the brain, colon, kidney, heart, lung and pancreas [12]. It can be concluded that CBS together with MST play a dominant role in the nervous system, while CSE with MST in the cardiovascular system [13–15].

CBS in its active form consists of four 63-kDa subunits, each of which contains three domains: PLP-binding domain, heme binding domain (N-terminal) and regulatory domain (C-terminal) [16]. The activity of CBS is influenced by many factors, both endogenous and exogenous [17]. The allosteric activator of CBS S-adenosylmethionine (SAM) can increase its activity [16,18]. Moreover, SAM was shown to modulate binding of other gasotransmiters NO and CO to the heme, and in this way it can also regulate enzymatic activity of CBS [19]. The activity of CBS is also modulated by S-glutathionylation [20], phosphorylation [21] and sumoylation [22]. Two sites of ubiquitination have been found in the CBS structure [23], and it can be suspected that also this common modification of proteins plays a role in the regulation of CBS activity.

CSE, just like CBS, is a tetramer but the molecular weight of its monomer is 45 kDa [12]. The regulation of CSE is not very well understood, yet. An *in vitro* study suggests sumoylation as a post-translational modification of CSE [24]. Expression of CSE is regulated by phosphorylation [25] and promotor methylation [26]. The increased expression of CSE was observed in some pathological conditions, i.e. in inflammation [27], oxidative stress [28,29] and in malnutrition [30].

In the case of MST regulation, the influence of reducing compounds such as Trx_red_ or DHLA is an important aspect [10]. Moreover, the MST/CAT pathway is inhibited by Ca^2+^ ions that limit H_2_S synthesis through CAT inhibition [31].

Another important source of H_2_S derives from microbiome in the human and animal gut. Sulfate-reducing bacteria (SRB) living in the oceans in the Archean have also been found in the mammalian gut, where they have a suitable anaerobic environment. SRB are ubiquitous members of the mammalian colon and include several types of bacteria with dominant genera *Desulfovibrio*. SRB can reduce sulfate to H_2_S non-enzymatically. Some species of SRB (namely *Desulfovibrio piger*) are also able to use sulfated glycans as a substrate [32]. It has been documented that a sulfate-rich diet results in increased H_2_S production in the colon of mice [33]. Besides SRB, several anaerobic bacterial strains living in the digestive tracts, including *Escherichia coli, Salmonella enterica, Clostridia* and *Enterobacter aerogenes* can metabolize cysteine to H_2_S, pyruvate and ammonia in reaction catalyzed by cysteine desulfhydrase [34,35]. Another possibility of H_2_S production by most of the above-mentioned and some other bacteria (*Klebsiella, Bacillus, Staphylococcus, Corynebacterium* and *Rhodococcus*) consist in the reduction of sulfite by sulfite reductase [36,37].

Some authors suggest that H_2_S production by the gut microbiota exceeds its endogenous formation from cysteine [38]; however, other researchers indicate an equal share of both sources [39]. It has been reported that the total sulfide concentration in the luminal content of the large intestine is in the range of millimolar concentration in mammals; however, less than 8% of the total sulfide exists in the free form due to a large binding capacity of feces [40,41]. Interestingly, studies performed with germ-free and conventional mice showed that the presence of the microbiota resulted in a higher H_2_S concentration in the plasma (0.03 nmol/mg protein vs. 0.009 nmol/mg protein) as well as in the intestinal tract [41]. Moreover, in the plasma of germ-free mice, a significant decrease (0.005 nmol/mg vs. 0.03 nmol/mg protein) in the level of bound sulfane sulfur, considered to be a way of H_2_S storage, was observed [42]. On the other hand, in most organs of germ-free animals, an increased cysteine level and a decreased activity of CSE were found [43]. These observations strongly support an important role of the microbiota in H_2_S production in the mammalian body as well as suggest an impact of the gut microflora on bioavailability of cysteine and activity of CSE. Flannigan et al. examined colonic H_2_S synthesis derived from bacteria and colonic tissue in healthy and colitic mice [39]. They found that approximately one half of the H_2_S in feces was derived from colonic cells. When the tissue is injured or during an inflammation, the production of H_2_S by colonic cells is markedly increased. Moreover, it was shown that the lack of PLP in the diet resulted in reduced fecal H_2_S level by approximately 50% [39]. It indicates that enzymatic formation of H_2_S by PLP-dependent enzymes (CBS and CSE) in colonic tissues is responsible for about a half of the total production of fecal H_2_S.

### Catabolism of H_2_S

H_2_S plays a signaling role only at physiological, relatively low concentrations. It is well known that in higher concentrations H_2_S is toxic as an inhibitor of complex IV of the respiratory chain, namely, cytochrome oxidase, and in this way, it impairs cell respiration [44,45]. In order to prevent this toxicity, bioavailability of H_2_S must be tightly regulated by its safe and nontoxic storage in the form of bound sulfane sulfur (see the next chapter) and by efficient catabolism. In mammals, H_2_S is oxidized mainly in mitochondria via the ‘sulfide oxidizing pathway’ with thiosulfate and sulfate being the end products of this pathway. Three enzymes are involved in the catabolism of H_2_S: sulfide quinone oxidoreductase (SQR), persulfide dioxygenase (ETHE1) and rhodanese also known as thiosulfate sulfurtransferase (TST) [46].

**Figure 5:**
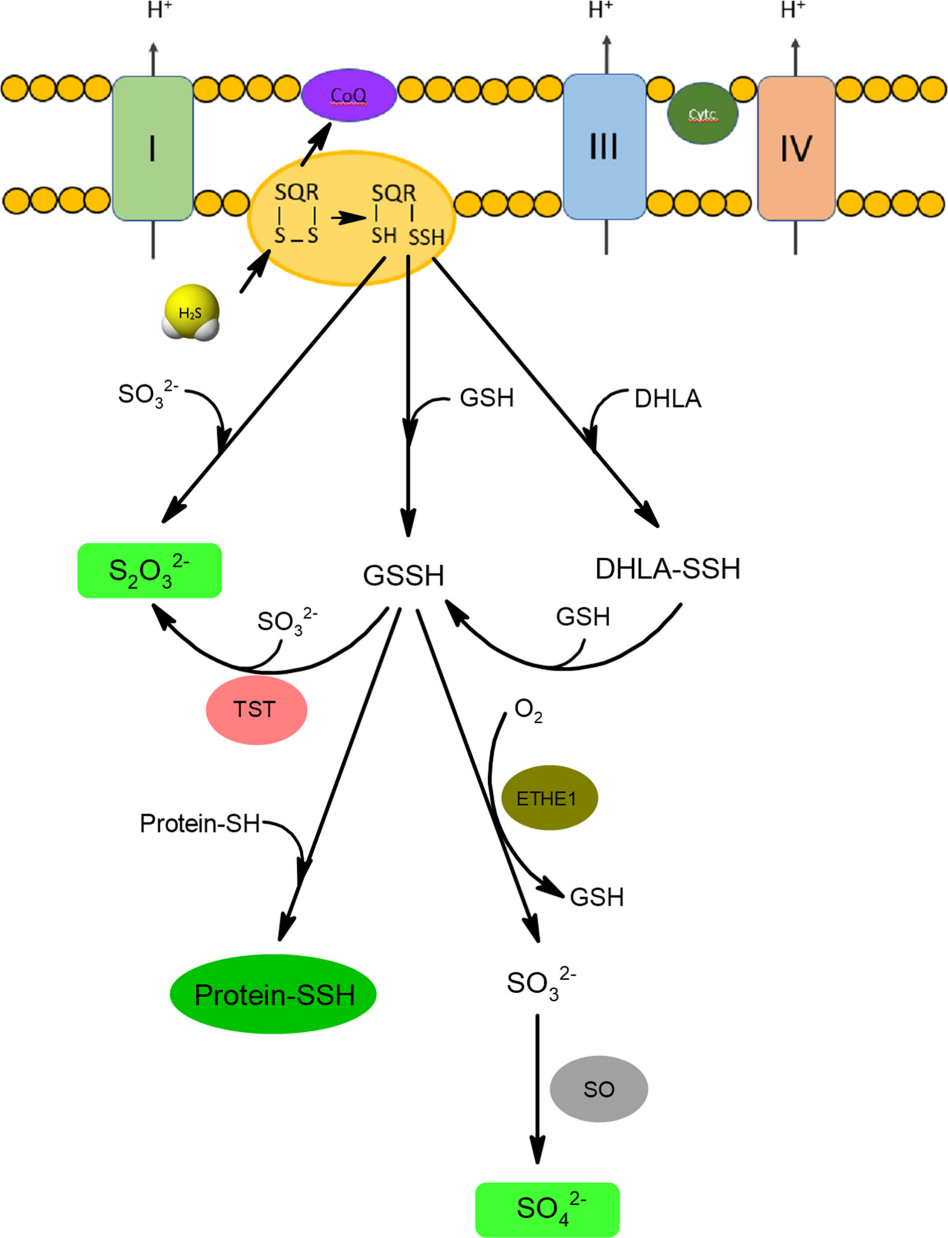
Mitochondrial oxidation of H_2_S H_2_S in the reaction with oxidized form of SQR forms persulfide that can be a donor of sulfur atom for glutathione (GSH), DHLA or sulfite anions forming appropriate persulfide forms: GSSH and DHLA-SSH or thiosulfate, respectively. Sulfur atom of GSSH can be next oxidized by persulfide dioxygenase (ETHE1) to sulfite (SO_3_^2−^) that is further oxidized to sulfate by sulfite oxidase (SO). Sulfur atom from GSSH can be also transferred to sulfite in the reaction catalyzed by rhodanese (TST) resulting in thiosulfate formation. Thiosulfate and sulfate anions are the main end products of H_2_S catabolism.

The H_2_S oxidizing pathway begins with the reaction catalyzed by SQR resulting in the formation of SQR persulfide (SQR-SSH) at one of its two Cys-SH residues [47–49]. SQR is a flavoprotein bound to the inner mitochondrial membrane, and while it is oxidized a noncovalently bound FAD is reduced and electrons are transferred to coenzyme Q contributing to the electron transport chain and ATP production. For this reason, H_2_S was regarded as the first inorganic substrate for oxidative phosphorylation comparable to succinate [44,50]. SQR is characterized by a low *K*_M_ value and high catalytic rate, which contributes to effective catabolism of H_2_S [48]. The sulfur atom from SQR-SSH can be transferred to glutathione (GSH) forming glutathione persulfide (GSSH) and next it can be oxidized to sulfite (SO_3_^2−^) by ETHE1 or transferred to sulfite by TST resulting in thiosulfate formation (Figure 5). Sulfite is further oxidized to sulfate by sulfite oxidase (SO). Sulfite can also be a direct acceptor of sulfur atom from SQR-SSH producing thiosulfate. Some authors suggested that endogenous reducing compounds other than GSH (i.e. DHLA) could accept the sulfur atom from SQR-SSH [51]. GSSH can also donate its sulfur atom to proteins forming protein persulfides [52].

**Figure 6:**
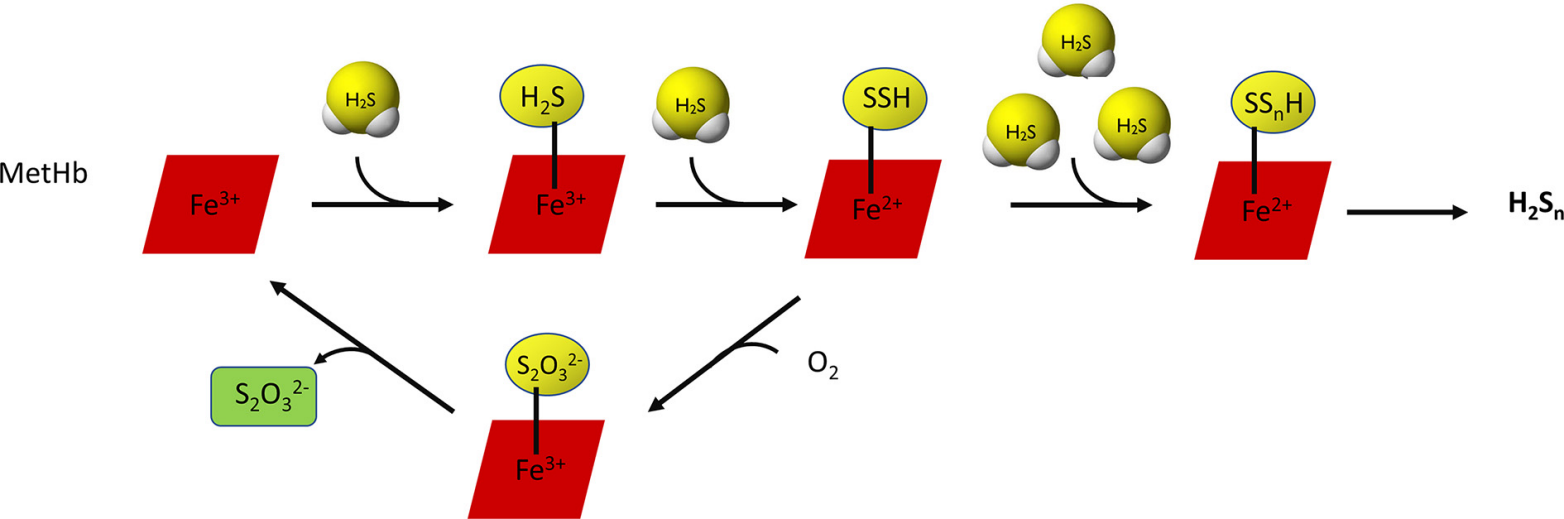
Hemoprotein-dependent oxidation of H_2_S with thiosulfate and inorganic polysulfide as end products Many molecules of H_2_S can bind to Fe^3+^ ion in heme (MetHb) forming persulfide or polysulfide chain. The persulfide can be oxidized in the presence of oxygen to thiosulfate. This pathway of H_2_S oxidation is important in erythrocytes that do not have mitochondria.

It is worth mentioning that H_2_S can also be oxidized by hemoproteins. In this case, the end products of H_2_S oxidation, i.e. thiosulfate and inorganic polysulfides, are bound to heme iron [53]. Initially, H_2_S binds to Fe^3+^ in heme (MetHb) and then another molecules of H_2_S combine forming persulfide or polysulfide chain. The persulfide can be oxidized in the presence of oxygen to thiosulfate, which can be released as the end product (Figure 6). Involvement of Hb in the H_2_S catabolism seems to be necessary in erythrocytes which do not have mitochondria. It has been documented that other proteins utilizing oxygen or H_2_O_2_ as an electron acceptor (i.e. catalase, superoxide dismutase, SOD) can also oxidize H_2_S to polysulfide [54,55].

## Persulfides (RSSH)

Persulfides (RSSH) in contrast to H_2_S contain sulfane sulfur atom bound to cysteine thiol. Sulfane sulfur is defined as a sulfur atom occurring in the 0 or -1 oxidation state covalently bound to another sulfur atom. In the RSSH structure, the sulfane sulfur atom is the outer sulfur with -1 oxidation state, while in elemental sulfur (S_8_) or in polysulfides (RSSSR) the oxidation state of sulfane sulfur atom is 0.

RSSH have aroused interest of researchers because they were proposed to mediate the H_2_S signaling. The search for the keyword ‘persulfide’ in the PubMed database yielded about 679 records, and since 1997 there has been a steady increase in interest in these compounds (Figure 1). A large body of evidence indicate that persulfides possess a similar physiological role as H_2_S. For this reason, RSSH have been shown to be responsible for some of the effects originally attributed to H_2_S. Indeed, H_2_S at higher concentrations is a toxic compound, while RSSH do not show such properties.

### General properties of RSSH

The chemical properties of RSSH are often compared with the RSH which are their thiol analogues. Replacing the -SH group in thiols with the -SSH group in persulfides affects energy of S-H bond, that is weaker by 92 kJ/mol in RSSH compared with RSH [56]. This energy difference will result in an increased stability and weaker toxicity of persulfide radicals (RSS**^●^**) in relation to thiyl radicals (RS^●^). The internal sulfur atom in RSS^●^ structure is stabilized by resonance effect [57] (reaction 2).

(2) $$\left[\text{R-}\underset{¨}{\ddot{\text{S}}}\text{-}\underset{¨}{\dot{\text{S}}}\text{:}⇄\text{R-}\underset{¨}{\dot{\text{S}}}\text{-}\underset{¨}{\ddot{\text{S}}}\text{:}\right]$$

Persulfides are more potent H atom donors than thiols, and similarly anionic perthiols (RSS^−^) proved to be better one-electron reductants compared with corresponding anionic thiol (RS^−^) [58,59]. It was confirmed by Koppenol and Bounds who based on thermodynamic data estimated that at pH 7.0 the electrode potential of RSS**^●^**/RSSH was +0.68V, while the potential for RS**^●^**/RSH was +0.96V [60]. For this reason, persulfides are considered to be stronger and more effective antioxidants than thiols. It was demonstrated that GSSH effectively reduced H_2_O_2_, while GSH or H_2_S in the same conditions was not sufficient in this reduction [61]. Recently, Li et al. showed by using resonance synchronous spectroscopy that GSSH was 50-fold more reactive than H_2_S relative to H_2_O_2_ at physiological pH (*k* values estimated for GSSH and H_2_S were 23.76 and 0.46 M^−1^s^−1^, respectively) [62]. The noticeable increase in the reducing capacity of persulfides relative to thiols can be attributed to the possibility of their tautomerization to thiosulfoxide form (reaction 3) [63].

(3) $$\text{R-S-SH}⇄\overset{\text{:}\underset{¨}{\ddot{\text{S}}}\text{:}}{\text{R-SH}}$$

Bianco et al. examined the ability of RSSH to act as one-electron reductants using an alkyl hydropersulfide model, namely, N-methoxycarbonyl penicillamine persulfide (MCP-SSH). They found that this persulfide was easily oxidized by the moderately strong one-electron oxidant 4-hydroxy-2,2,6,6-tetramethylpiperidine 1-oxyl (TEMPOL) to TEMPOL-OH, while the corresponding thiol was not. MCP-SSH was also efficiently oxidized by K_3_Fe(CN)_6_ and Fe(III) of myoglobin, while in the case of the corresponding thiol, reduction of myoglobin Fe(III) did not occur and reduction of K_3_Fe(CN)_6_ was less than 50% of that with MCP-SSH [64]. Moreover, a study performed on A549 cells revealed that the cells with overexpression of CSE responsible for persulfide formation showed a better viability after treatment with H_2_O_2_ than those with normal CSE expression [61]. All these facts clearly confirm that persulfides are much more powerful reductants compared with corresponding thiols. However, it should be noted that all the above-mentioned facts were derived based on *in vitro* experiments using comparable concentrations of thiols and persulfides. However, the concentrations of persulfides present in cells are much lower than thiol concentrations what means that persulfides are even more reactive than thiols, in physiological conditions antioxidant reactions involving thiols, especially GSH, predominate due to considerably higher concentration of GSH than GSSH.

Persulfides are characterized by a higher acidity when compared with thiols (reactions 4 and 5) [65].

(4) $$\text{RSSH}⇄{\text{RSS}}^{-}+{\text{H}}^{+}$$

(5) $$\text{RSH}⇄{\text{RS}}^{-}+{\text{H}}^{+}$$

The p*K*_a_ value for 2-[3-aminopropyl)amino]ethane persulfide was estimated at 6.2, whereas the p*K*_a_ for the corresponding thiol was 7.6 [58]. On the other hand, based on computational calculation the p*K*_a_ of cysteine persulfide (CysSSH) was estimated at 4.3, while the p*K*_a_ of CysSH is known to be 8.3 [66]. The p*K*_a_ value of glutathione persulfide (GSSH) is a little controversial because Li et al. using resonance synchronous spectroscopy estimated it at 6.9 [62], while Benchoam et al. in the reaction with mBrB determined it as 5.45 [67]. The p*K*_a_ of GSH is assumed to be 8.9, so independently of the difference in the estimated values of p*K*_a_ for GSSH, persulfides possess much stronger acidic character. Based on these data, it can be concluded that at physiological pH anionic perthiol species (RSS^−^) dominate over RSSH forms, whereas thiols predominantly exist in the protonated form (RSH).

### Nucleophilicity of RSSH

Persulfides, like thiols can act as nucleophiles (reactions 6 and 7).

(6) $${\text{RSS}}^{-}+{\text{E}}^{+}⇄\text{RSSE}$$

(7) $${\text{RS}}^{-}+{\text{E}}^{+}⇄\text{RSE}$$

**Figure 7:**
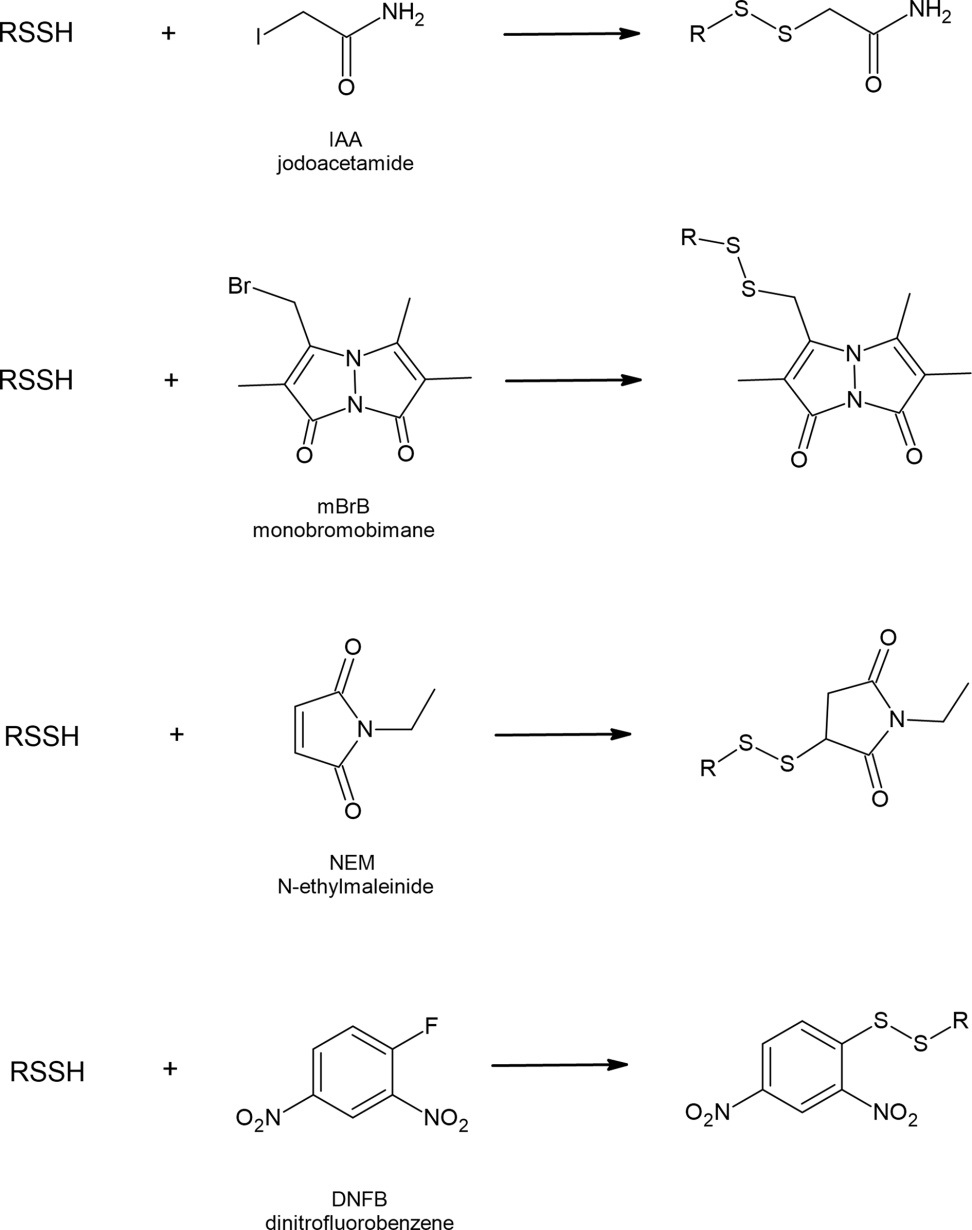
Reactions of RSSH with popular electrophilic alkylating agents

They react with electrophilic alkylating agents, such as iodoacetamide (IAA), monobromobimane (mBrB), N-ethylmaleimide (NEM) and dinitrofluorobenzene (DNFB) yielding appropriate disulfides (Figure 7). Thiols in the same reactions form respective thioethers. Reactions with electrophiles are also used for persulfide determination; however, other new methods have been developed [68,69]. It has also been documented that persulfides can efficiently react with 8-nitroguanosine 3′,5′-cyclic monophosphate (8-nitro-cGMP), an endogenously occurring weak electrophile acting as a second messenger in redox signaling [61].

Persulfides are considered to be stronger nucleophiles than corresponding thiols. Benchoam et al. studied the kinetics of GSSH reactions with three various electrophiles and compared the data with parameters for GSH. The results indicate that at physiological pH the reactivity of GSSH toward mBrB, peroxynitrite and H_2_O_2_ is enhanced, when compared with GSH. According to the literature the estimated rate constants for GSSH and GSH are: 7.02 × 10^3^ and 208 M^−1^s^−1^; 7.5 and 0.42 M^−1^s^−1^; 1.25 × 10^5^ and 1360 M^−1^s^−1^ toward mBrB, H_2_O_2_ and peroxynitrite, respectively [67,70,71]. The increased nucleophilicity of persulfide compared with thiol has also been documented in the case of albumin persulfide in reaction with disulfide 4-4′-dithiodipyridine. Cuevasanta et al. reported the following second-order rate constants: 1.7∙10^4^ M^−1^ s^−1^ for HSA-SSH and 7.6 × 10^2^ M^−1^ s^−1^ for HSA-SH determined at pH 7.4 and at 25°C [66]. It can be concluded that persulfides react faster than thiols because of increased availability of the anion and so called alfa-effect (increased reactivity of an atom caused by the presence of unshared pairs of electrons in the adjacent atom). A similar effect has been observed for other pairs of compounds with nucleophilic character (NH_2_NH_2_/NH_3_ and HOO^−^/HO^−^) [72]. Strong nucleophilic character of RSSH makes them able to be used as a kind of defense against toxins with electrophilic nature. It has been documented that an electrophilic metabolite of acetaminophen N-acetyl-p-benzoquinone imine (NAPQI) reacts with CysSSH or GSSH forming mixed disulfides NAPQIH_2_-SSCys or NAPQIH_2_-SSG, respectively [73]. Sulfane sulfur-containing persulfides can also react with other electrophiles, such as methylmercury (MeHg) [74] or 1,4-naphthoquinone (1,4-NQ) [75] yielding various sulfur adducts that may be advantageous in toxicology [76].

### Electrophilicity of RSSH

Regardless of the nucleophilic properties, persulfides when protonated possess also electrophilic character what distinguishes them from thiols. Nucleophiles can attack the outer sulfur atom of persulfides that leads to thiol formation; on the other hand, nucleophile attack on the inner sulfur atom produces H_2_S. The general reaction scheme and reactions of RSSH with popular nucleophiles that attack the outer sulfur atom are presented below in part A, whereas the reaction representing the attack of nucleophile on the inner sulfur atom is presented in part B.

**A**

$$\mathrm{RSSH}+{\mathrm{Nu}}^{-}⇄{\mathrm{RS}}^{-}+\mathrm{NuSH}$$

(8) $$\text{RS}S\text{H}+{\text{CN}}^{-}⇄{\text{RS}}^{-}+\text{H}S\text{CN}$$

(9) $$\text{RS}S\text{H}+{\text{SO}}_{3}^{2-}⇄{\text{RS}}^{-}+\text{H}S{\text{SO}}_{3}^{2-}$$

(10) $$\text{RS}S\text{H}+{\text{OH}}^{-}⇄{\text{RS}}^{-}+\text{H}S\text{OH}$$

(11) $$\text{RS}S\text{H}+{{\text{R}}^{\prime }}_{3}\text{P}⇄\text{RS}H+{{\text{R}}^{\prime }}_{3}\text{P}=S$$

(12) $$\text{RS}S\text{H}+{\text{R}}^{\prime }{\text{S}}^{-}⇄{\text{RS}}^{-}+{\text{R}}^{\prime }\text{S}\mathrm{SH}$$

**B**

$$\mathrm{RSSH}+{\mathrm{Nu}}^{-}⇄{\mathrm{HS}}^{-}+\mathrm{RSNu}$$

(13) $$\text{R}S\text{SH}+{\text{R'S}}^{-}⇄\text{RSSR'}+{\text{HS}}^{-}$$

The (reaction 8) between persulfide and cyanide yielding thiocyanate is used as the oldest method of sulfane sulfur detection called cyanolysis [77]. In this method, thiocyanate formed by cyanide attack on sulfane sulfur atom reacts then with ferric ions leading to the formation of a colored complex that can be assayed spectrophotometrically. Transfer of reactive sulfane sulfur atom from persulfides to nucleophilic acceptors, such CN^−^, sulfite or other thiols (RS^−^) can be catalyzed in biological conditions by rhodanese (TST). The reaction of persulfide with thiol, in which the outer sulfur atom is the target of RS^−^ anion attack, leads to transpersulfidation reaction, where a new persulfide is formed (reaction 12). This process occurs when enzymes involved in H_2_S and sulfane sulfur metabolism (TST, MST, SQR) accept the sulfur atom forming their intermediate persulfide and then transfer sulfur atom to other acceptors. Moreover, the transpersulfidation process plays an important role in creation of protein -SSH groups. Low molecular weight persulfides, especially GSSH, are regarded as responsible for protein persulfidation [52].

**Figure 8:**
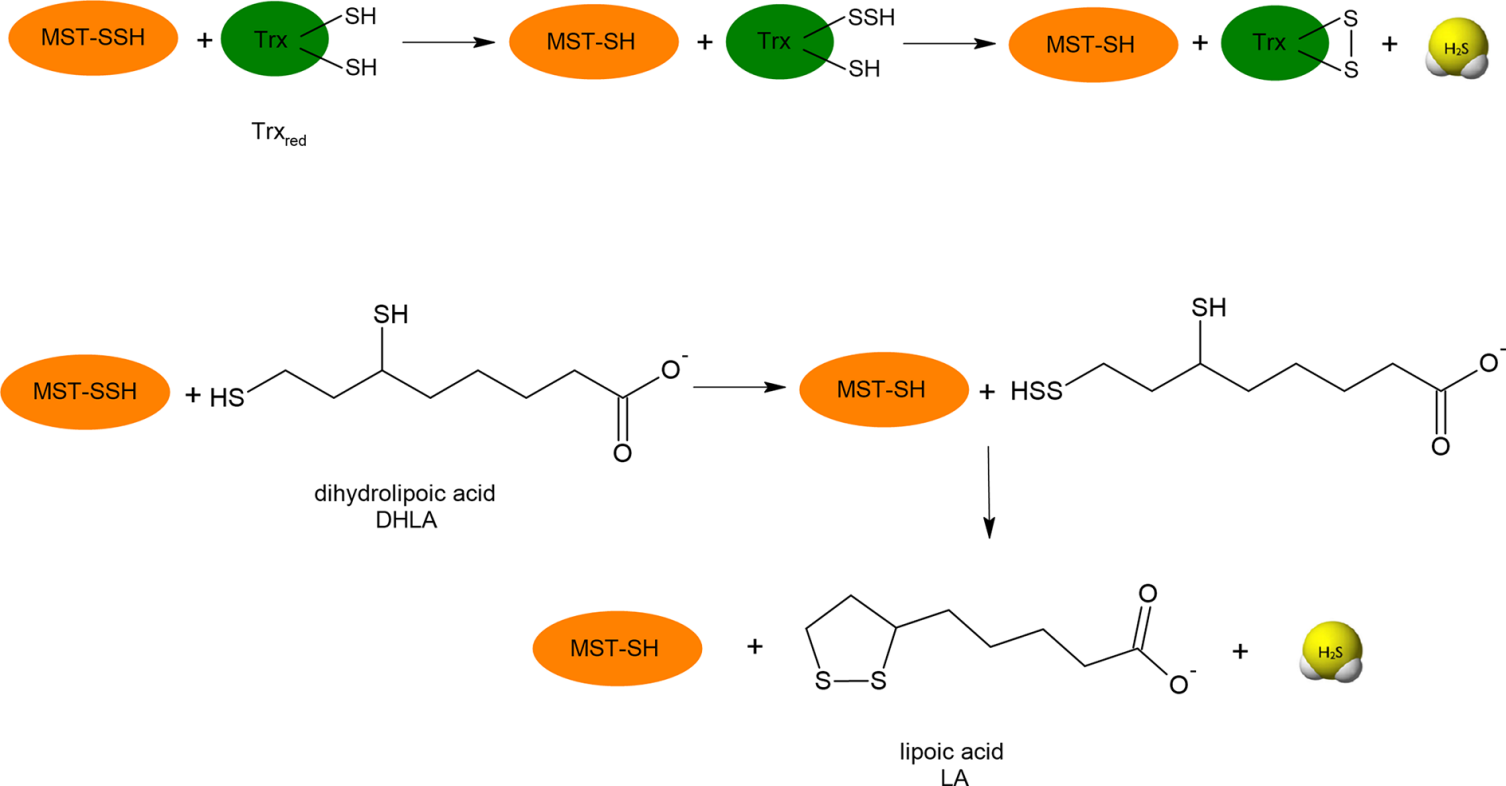
Endogenous dithiols: Trx and lipoic acid (LA) are responsible for release of H_2_S from MST persulfide (MST-SSH) DHLA (reduced form of lipoic acid)

On the other hand, when the inner sulfur atom in the persulfide is the target of RS^−^ anion attack, in this reaction H_2_S is released and mixed disulfide is formed (reaction 13) [65,78]. This mechanism is preferred by small, low molecular weight persulfides that in reaction with thiol form mainly intermolecular disulfide bonds and release H_2_S. In the case of MST persulfide (MST-SSH), it has been documented that H_2_S can be released only by the small protein Trx_red_ or DHLA. Both these compounds as dithiols have a close pair of -SH groups and during the reaction one of the two thiols forms a persulfide (so it is a transpersulfidation reaction), which is then attacked at the inner sulfur by the vicinal thiol leading to the formation of intramolecular disulfide of Trx_ox_ or LA, respectively (Figure 8) [79]. It seems that the reduction of protein persulfides by dithiols can also occur in other persulfidated proteins. It has been demonstrated that the activity of yeast aldehyde dehydrogenase (ALDH) inhibited by RSS was recovered much better by DHLA and dithiothreitol (DTT) than by GSH [80].

As mentioned earlier, persulfides show an electrophilic character when exist in their protonated form (RSSH). Considering that based on p*K*_a_ value at physiological pH conditions in the cells anionic perthiol species (RSS^−^) shall prevail over RSSH forms, it seems that in biological processes nucleophilic character of persulfides dominates over electrophilic.

### Synthesis and endogenous occurrence of RSSH

Reactivity and properties of persulfides have been studied using various low molecular weight compounds or protein models. The synthesis of a few organic persulfides, including benzyl persulfide (BnSSH), trityl persulfide (TrtSSH) and adamantyl persulfide (AdSSH) has been described [81–83]. Details of the synthesis of these compounds from acetyl sulfenyl chloride and appropriate thiols have been given in the papers mentioned above. Using these persulfide models, some conclusions regarding structure–reactivity relationship can be drown. For example, it has been established that acidic persulfides are more prone to decomposition than the less acidic ones [82]. Moreover, these studies have also revealed that persulfides can release H_2_S under the influence of reducing agents [83].

Apart from those model of persulfides which rather are not present in biological conditions, GSSH and CysSSH are the well-known and widespread low molecular weight persulfides in the cells. The most popular, standard method to generate low molecular weight persulfides, such as GSSH or CysSSH, *in vitro* is the reaction between a disulfide (GSSG or CysSSCys) and Na_2_S or between sulfenic acids (RSOH) and Na_2_S [66,67,78,80,84]. This technique is fast and easy, however, it has some weak points, namely it requires high concentrations of the used disulfides and often thiol and sulfide can be found in the reaction mixture apart from persulfides.

Both biologically important persulfides, GSSH and CysSSH, just like other compounds of this kind, are quite unstable what makes them a difficult objects to study. The main products of decomposition of persulfides include thiols, polysulfides and elemental sulfur (S_8_). H_2_S is not a direct product of decomposition, however, in the presence of thiols it can also be formed [78]. The reactivity of biological persulfides CysSSH and GSSH should be similar to the reactivity of other low molecular weight persulfides (RSSH) mentioned above [85]. A study by Benchoam et al. examining the acidity and nuclephilic reactivity of persulfides performed using GSSH confirmed it [67].

**Figure 9:**
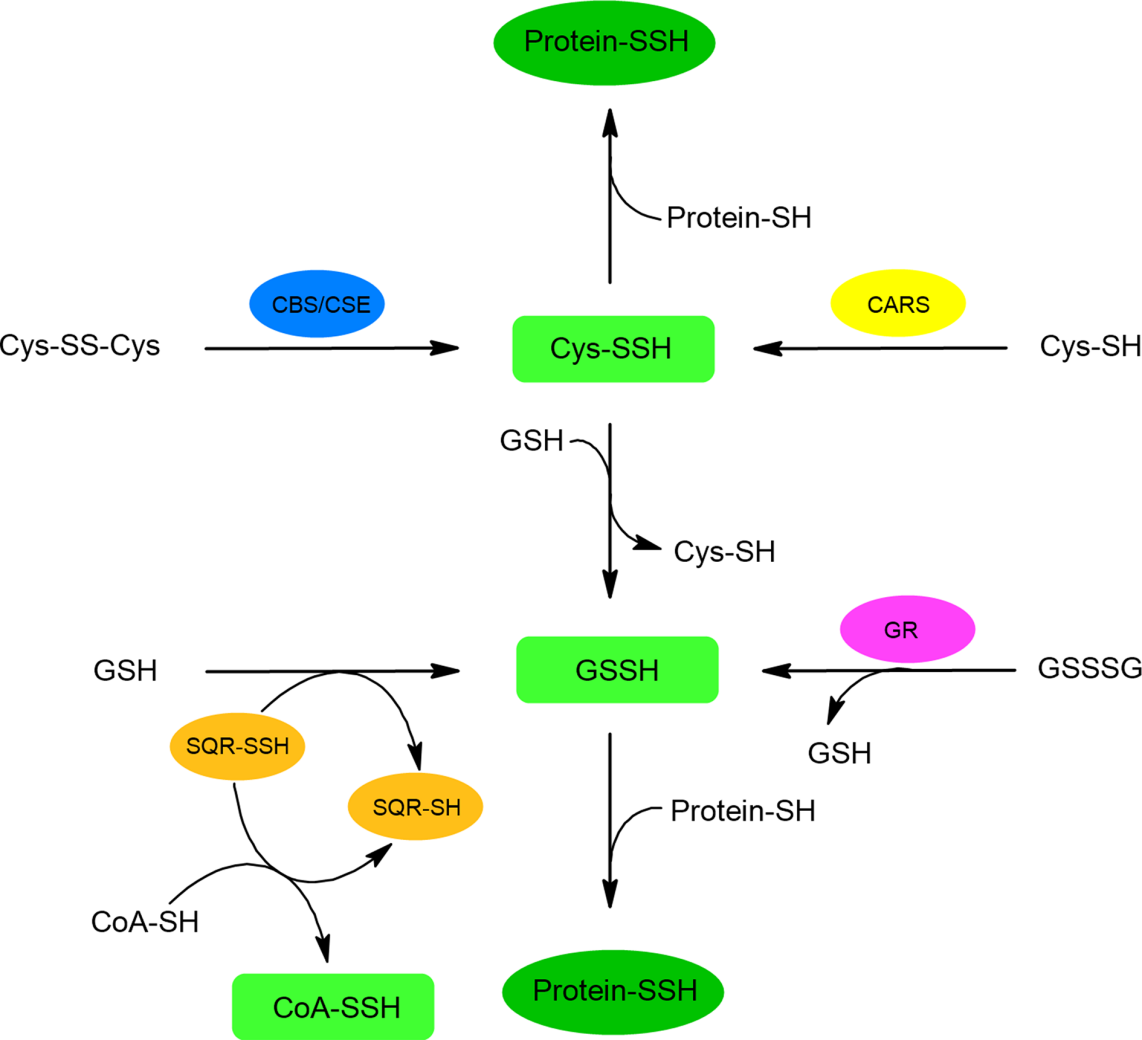
Endogenous synthesis of low molecular weight and protein persulfides Biological persulfides are produced mainly in reactions catalyzed by CBS and CSE that convert cystine (CysSSCys) to cysteine persulfide (CysSSH, thiocysteine). CARS catalyzes the formation of CysSSH from CysSH during translation process. SQR, involved in mitochondrial oxidation of H_2_S, produces glutathione persulfide (GSSH) by transferring sulfane sulfur atom to GSH. SQR can also form persulfide of CoA-SH. Moreover, GR can produce GSSH from glutathione polysulfide (GSSSG).

*In vivo* persulfides are produced mainly in reactions catalyzed by CBS/CSE and SQR; however, glutathione reductase (GR) can also be responsible for GSSH formation (Figure 9). CBS and CSE can convert cystine (CysSSCys) to CysSSH called also thiocysteine [86]. Thiocysteine can give its sulfane sulfur atom to GSH, the concentration of which in cells is high, forming GSSH (transpersulfidation). As mentioned above, SQR is an enzyme involved in H_2_S oxidation in mitochondria and it can create GSSH by transferring its sulfane sulfur atom to GSH (Figure 9) [87]. GSSH can also be produced from glutathione polysulfide in reaction catalyzed by GR [61]. CysSSH and GSSH have been detected in various cell cultures including HEK293 [88], A549, SH-SY5Y, C6 [61] and HeLa [57].

Recently, it has been found that the formation of another important endogenous thiol CoA-SH in its persulfide form can also be mediated by SQR. A study by Landry et al. using human SQR supported the conversion of CoA-SH to CoA-SSH indicating that this enzyme can utilize CoA-SH as an alternative sulfur acceptor. CoA-SSH formed in this reaction inhibits butyryl-CoA dehydrogenase (ACADS) and butyrate oxidation [89,90]. According to literature data, the GSSH concentration in animal tissues is in a range 10–100 μM in most organs, while the highest concentration of approximately 150 μM was found in the brain [61,91]. The level of CysSSH has also been estimated in μM range and the level of both endogenous persulfides (CysSSH and GSSH) was dependent on the availability of sulfur compounds (e.g. methionine) in the diet [61] as well as on the expression of enzymes involved in their synthesis [61,88]. The presence of GSSH and CysSSH has also been demonstrated in various human tissues, including plasma [61,92], lung resident cells and epithelial lining fluid [93], sputum [94], aqueous and vitreous humor [95], tear, saliva, and nasal discharge [96]. Information concerning enzymes involved in physiological formation of persulfides is summarized in Table 1.

### Protein persulfides

Protein persulfides (protein-SSH) are formed mainly by reaction with low molecular weight persulfides, such as GSSH or CysSSH that means in a transpersulfidation reaction. It seems that GSSH is the most widespread low molecular weight persulfide, and it plays a dominant role in the protein persulfide forming reaction. In this process called protein persulfidation or sulfuration, the protein CysSH residue is oxidized to CysSSH yielding protein persulfide. This process is considered as a reversible, post-translational, covalent modification of proteins which plays an important regulatory and protective role [52,97–99]. Regarding the mechanism of protein persulfidation, two pathways are possible: protein CysSH reacts with RSSH or another compound containing sulfane sulfur atom or protein CysSH oxidized to sulfenic acid, disulfide, mixed disulfide or nitrosothiol is persulfidated with H_2_S [100].

More recently, it has been documented that protein persulfides are formed *in vivo* also in reactions catalyzed by cysteine persulfide synthase called cysteinyl-tRNA synthetase (CARS). This enzyme catalyzes the formation of CysSSH from CysSH as a substrate during translation process [88]. CysSSH-tRNA formed by CARS can be a substrate for protein synthesis in ribosomes and can lead to persulfidation of newly synthesized proteins. It means that protein persulfidation can be a post-translational modification and a translation-coupled process. It seems that both mechanisms play important roles in maintenance of proper physiological level of protein persulfidation.

## Polysulfides

Polysulfides including inorganic hydrogen polysulfides (H_2_S_*n*_, *n*>2) and organic polysulfides (RS_*n*_H and RS_*n*_R, where *n*>2), just like persulfides, contain reactive sulfane sulfur atom in their structure. This kind of RSS became the focus of interest after it has been revealed that they exist endogenously and participate in RSS redox signaling. The great interest in these forms is evidenced by the fact that the search for the keyword ‘polysulfide’ in PubMed currently results in over 2900 records (Figure 1). In many cases, polysulfides are being considered together with persulfides since both H_2_S-related RSS are linked to some physiological and pathological processes. Moreover, some methods used for persulfide and polysulfide determination do not distinguish between these two kinds of RSS. However, from chemical point of view, there are a lot of points that differ these two kinds of RSS and this is why polysulfides should be described separately. In recent years, several new methods have been developed to selectively determine polysulfides in biological samples [101–104].

### Synthesis of polysulfides

**Figure 10:**
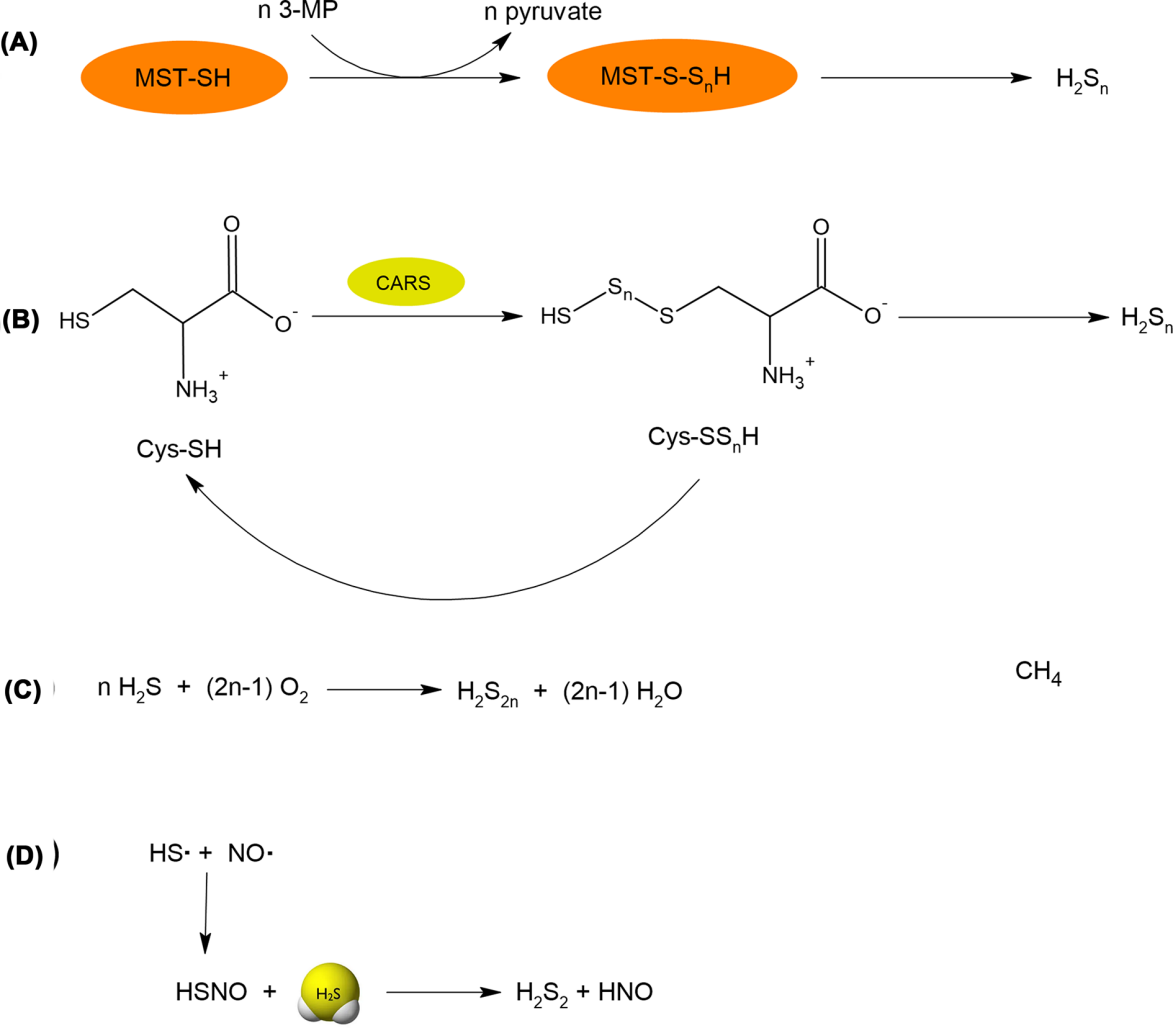
Reactions responsible for polysulfide synthesis (**A**) Inorganic polysulfides are produced by MST, which uses 3-MP as a substrate. (**B**) CARS using L-cysteine as a substrate effectively produces cysteine hydropolysulfides (CysSS_*n*_H), which can release inorganic polysulfides. (**C**) An example of nonenzymatic generation of inorganic polysulfides by simple oxidation of H_2_S. (**D**) Inorganic polysulfides can also be produced in reaction of H_2_S with thionitrous acid (HSNO) formed in reaction of two radicals (NO^•^ and HS^•^).

Biosynthesis of inorganic and organic polysulfides is closely related to the synthesis of persulfides. Inorganic polysulfides were first detected in mouse brains by Kimura et al. [105]. Their studies revealed the presence of H_2_S_3_ in the cytosol of brain cells. The enzyme responsible for the production of these polysulfides was recognized as MST, which uses 3-MP as a substrate (Figure 10A). Besides H_2_S_3_, H_2_S_2_ and H_2_S_5_ were also identified in the studied brain samples; however, they were minor products [105]. The second enzyme that can contribute to the production of polysulfides is CARS mentioned above in the persulfide section. It was shown by Akaike et al. that CARS could effectively catalyze the production of not only Cys-SSH but also Cys-S_*n*_SH using Cys-SH as a substrate (Figure 10B) (Table 1) [88]. Cys-S_*n*_SH formed in this reaction either in the free form or incorporated in protein structure is an example of organic polysulfides; however, on the other hand, it can be a precursor of inorganic H_2_S_*n*_.

Inorganic polysulfides can also be generated non-enzymatically by oxidation of H_2_S (Figure 10C) [106]. This reaction is rather slow, that is why it is not an important source of polysulfides inside the cells. However, this reaction is often responsible for contamination of commercially available sulfides by inorganic polysulfides. Non-enzymatic reactions generating H_2_S_2_ include also the reaction between radical forms of two gasotransmiters: H_2_S and nitric oxide (NO). Thionitrous acid (HSNO) formed in this reaction can next react with the H_2_S molecule leading to H_2_S_2_ and nitroxyl (HNO) (Figure 10D) [107]. Since NO and H_2_S both occur in the cells, this reaction can be at least partially responsible for the production of polysulfide in the mammalian body.

Polysulfides can also be generated in heme-catalyzed reactions. As presented in Figure 5, H_2_S can be oxidized by heme of Mb and Hb or other hemoproteins to polysulfides bound to iron atom [53]. Free inorganic polysulfides can be next released spontaneously under the influence of endogenous reductors. Olson et al. found that cytosolic form of SOD could catalyze the oxidation of H_2_S yielding H_2_S_2_, H_2_S_3_ and H_2_S_5_ [55]. Another pathway of polysulfide formation involves the reaction between H_2_S and RSSR, which results in the production of RSSSR together with RSH and RSSH [108].

### Properties of polysulfides

Polysulfides are rather unstable species. According to the results of experiments performed with glutathione polysulfide (GSS_*n*_SG) [88] and alkylated polysulfides (RSS_*n*_SR) [109], sulfur-sulfur bonds in such compounds are susceptible to hydrolysis at physiological and alkaline conditions. The hydrolysis of polysulfides results in formation of reduced thiols and polysulfenic acids or hydropolysulfides and sulfenic acids (reactions 14-19).

(14) $${\text{RSS}}_{\text{n}}\text{SR}(\text{n}>1)+{\text{H}}_{2}\text{O}⇄\text{RSH}+{\text{RS}}_{\text{n}}\text{SOH}$$

(15) $${\text{RSS}}_{\text{n}}\text{SR}(\text{n}>1)+{\text{H}}_{2}\text{O}⇄{\text{RSS}}_{\text{n}}\text{H}+\text{RSOH}$$

(16) $$\text{RSSR}+{\text{H}}_{2}\text{O}⇄\text{RSH}+\text{RSOH}$$

(17) $$\text{HSSR}+{\text{H}}_{2}\text{O}⇄{\text{H}}_{2}\text{S}+\text{HSOH}$$

(18) $${\text{HS}}_{\text{n}}\text{H}+{\text{H}}_{2}\text{O}⇄{\text{H}}_{2}{\text{S}}_{\text{n}-1}+\text{HSOH}$$

(19) $${\text{HS}}_{\text{n}}\text{H}+{\text{H}}_{2}\text{O}⇄{\text{H}}_{2}\text{S}+\frac{\left(\text{n}-1\right)}{8\;{\text{S}}_{8}}$$

This high susceptibility of polysulfides (RSS_*n*_SR, *n*≥1) to hydrolysis distinguishes them from disulfides (RSSR), which undergo a hydrolysis reaction only under strong alkaline conditions. The alkaline hydrolysis of RSSR leads to production of reduced thiol (RSH) and sulfenic acid (RSOH) [104,110]. At neutral pH, the equilibrium of the reaction of RSSR hydrolysis is shifted to the left, so disulfides at physiological pH rather do not undergo hydrolysis.

Inorganic polysulfides (H_2_S_*n*_) are characterized by low stability at neutral pH. Moreover, it has been documented that various salts of inorganic polysulfides often used as polysulfide donors, differ in stability. For example, Na_2_S_2_ and Na_2_S_4_ were found to be relatively stable compounds but Na_2_S_3_ can readily decompose forming a mixture of Na_2_S_2_ and Na_2_S_4_ [111]. As long as the first product of this decomposition is consistent with the (reaction 18) (sulfenic acid is the second product), the formation of Na_2_S_4_ may be surprising. It can be explained by taking into account the suggestions of some authors that H_2_S_*n*_ can also decompose to H_2_S and elemental sulfur according to (reaction 19) [102]. In this way, HS^−^ ion and elemental sulfur present in the solution as a result of H_2_S_*n*_ decomposition can combine forming a new polysulfide (as Na_2_S_4_, more stable than Na_2_S_3_).

It has been shown that various alkylating reagents including IAA, NEM, mBrB or dimedone can enhance the hydrolysis of polysulfides [104,112]. During the hydrolysis, there is some equilibrium between RSS_*n*_SR and its products, i.e. RSSH and RSSOH. Alkylating agents reacting with RSSOH or RSSH shift this equilibrium to the right leading to enhanced decomposition of RSS_*n*_R. Interestingly, it was revealed that such alkaline hydrolysis of polysulfides could be significantly prevented by hydroxyl group-containing compounds such as β-(4-hydroxyphenyl)ethyl iodoacetamide (HPE-IAA), tyrosine or serine [104,112]. This observation suggests that moieties of naturally occurring amino acids containing hydroxyl groups (tyrosine and serine) may be involved in maintaining the polysulfide stability and in the regulation of their homeostasis.

**Figure 11:**
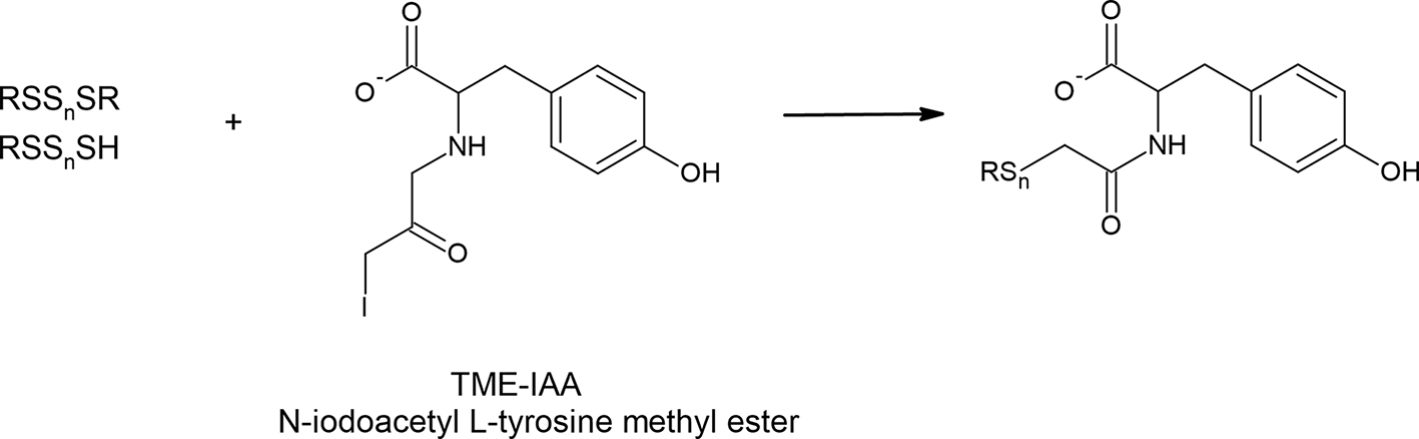
N-iodoacetyl L-tyrosine methyl ester (TME-IAA) is an alkylating agent which reacts with polysulfides and protects them from hydrolytic degradation

The finding that the hydroxyl group-containing agents efficiently protect polysulfides from hydrolytic degradation led the Akaike’s research group to develop the new alkylating agent namely, N-iodoacetyl L-tyrosine methyl ester (TME-IAA) (Figure 11). This compound specifically traps and stabilizes polysulfides and protects them against hydrolysis, and used with mass spectrometry allows for successful polysulfide analysis [103,104]. Kasamatsu et al. noted a loss of nearly 40% of glutathione tetrasulfide after 1 h of incubation at physiological pH while in the presence of mBrB, the total loss reached nearly 70% of the initial amount of tetrasulfide. In contrast, in the presence of TME-IAA this degradation was not observed [103]. The same authors reported higher amounts of various polysulfides and smaller amounts of sulfides in mouse tissues when they were detected with TME-IAA compared with amounts determined with HPE-IAA. It confirms that the determined level of RSS, especially polysulfides and H_2_S can be influenced by specific alkylating agents.

## Reactive sulfur species in diseases

As RSS play an important regulatory and protective role, it is obvious that disruption of their homeostasis can lead to some pathological conditions. It has been quite well documented that many diseases are associated with a disturbance of the endogenous RSS pool, most often with its decrease. Development of specific methods for the determination of H_2_S and especially persulfides and polysulfides enabled the study of their significance in some diseases. However, it is worth mentioning that an assay of any kind of RSS is a challenge due to their reactivity and instability. Many factors, including, pH, oxygen content, presence of oxidants or reductors can influence the final assay results. Moreover in the case of persulfides, the problem with appropriate stable standards exists. In the case of poly- or persulfides there are only limited studies reporting the levels of these RSS in physiological conditions (Table 2).

**Table 2:** Disturbances of RSS level and enzymes expression in some pathologies

Pathology	RSS or enzyme	Tissue	Health	Disease	Reference
**Cardiovascular disease**					
Spontaneous hypertension	H_2_S	Rat plasma	48 μM	20 μM^*^	[116]
Portal hypertension	H_2_S	Human plasma	43 μM	42 μM Child-Pugh score A 33 μM^*^ Child-Pugh score B 22 μM^*^ Child-Pugh score C	[115]
**Chronic kidney disease (CKD)**					
Hemodialysis	H_2_S	Human plasma	≈15 μM	≈11 μM^*^	[119]
CKD (non-dialysed)	H_2_S	Human plasma	14 μM	7 μM^*^	[43]
Hemodialysis and diabetic nephropathy	H_2_S	Human plasma	57 μM	23 μM^*^ CKD + diabetic nephropathy 29 μM^*^ μM CKD	[120]
**Metabolic disorders**					
Overweight	H_2_S	Human plasma	39 μM	22 μM^*^	[122]
Overweight and DM2	H_2_S	Human plasma	39 μM	10 μM^*^	[122]
DM2	H_2_S	Human plasma	130 μM	110 μM^*^	[125]
Streptozotocin-induced diabetes	H_2_S	Rat plasma	78 μM	45 μM^*^	[125]
Fructose-induced diabetes	H_2_S	Mice plasma	≈1.3 μM	≈1.0 μM^*^	[126]
Fructose-induced diabetes	H_2_S	Mice heart	≈0.20 μM	≈0.14 μM^*^	[126]
Streptozotocin-induced diabetes	H_2_S CSE expression CBS expression	Rat liver	≈37 μM	≈65 μM^*^ ↑^*^ ↑^*^	[128]
	H_2_S CSE expression CBS expression	Rat pancreas	≈30 μM	≈45 μM^*^ ↑ ↑^*^	[128]
Diabetic retinopathy	CysSSH	Human aqueous humor	0.14	0.28^*^μM	[95]
Diabetic retinopathy	CysSSH	Human vitreous humor	0.07	0.18^*^μM	[95]
Diabetic retinopathy	GSSSG	Human aqueous humor	0.07	0.19^*^ μM	[95]
**Neurodegenerative diseases**					
Mouse model of Parkinson’s disease	H_2_S	Mice striatum	≈ 39 μM	≈ 29 μM^*^	[129]
Mouse model of Parkinson’s disease	H_2_S	Mice plasma	≈ 22 μM	≈ 8.5 μM^*^	[129]
Parkinson disease	Parkin persulfides	Human striatum	control value	40% of the control value	[154]
Alzheimer disease	H_2_S	Human plasma	45 μM	34 μM^*^	[130]
Alzheimer disease	H_2_S	Human plasma	below 1 μM^A^	below 1 μM^A^	[131]
Alzheimer disease	Bound sulfur (persulfides)	Human plasma	below 1 μM^A^	above 1 μM^A^ ↑^*^	[131]
Amyotrophic lateral sclerosis (ALS)	H_2_S	Human cerebrospinal fluid	3.6 mg/l	7.5 mg/l^*^	[132]
Down syndrome	Thiosulfate	Human urine	3.3 mmol/mol creatinine	7.6 mmol/mol creatinine^*^	[133]
Rat model of Down syndrome	CBS expression	Brain		↑^*^	[135]
**Respiratory diseases**					
Asthma in children	H_2_S	Human serum	53 μM	44 μM	[137]
Asthma	H_2_S	Human serum	152 μM	280 μM^*^ non-severe 283 μM^*^severe	[138,142]
Asthma	H_2_S	Human sputum	11 μM	27 μM^*^ non-severe 28 μM^*^ severe	[138,140]
COPD	H_2_S	Human sputum	20 μM smokers 12 μM non-smokers	32 μM^*^ stable COPD 50 μM^*^ acute exacerbation COPD	[141]
COPD	H_2_S	Human serum	91 μM smokers 91 μM non-smokers	149 μM^*^ stable COPD 49 μM acute exacerbation COPD	[141]
COPD	GSSH	Bronchial epithelial	71 nmol/g protein	51 nmol/g protein^*^	[93]
	Bronchial epithelial CysSSH		31 nmol/g protein	21 nmol/g protein^*^	[93]
Asthma–chronic obstructive pulmonary disease overlap	Persulfides and polysulfides	Human sputum	16 arbitrary units (AU)	5.2^*^ arbitrary units (AU)	[94]
**COVID-19**					
COVID-19	H_2_S	Human plasma	0.31 μM	0.19^*^ μM	[144]
COVID-19	Bound sulfur	Human plasma	≈ 0.50 μM	≈ 0.62 μM NS	[144]
**Osteoarthritis**					
Osteoarthritis	H_2_S MST expression CBS expression	Human cartilage	0.46 nmoles/g of cartilage	0.06 nmoles/g of cartilage^*^ ↓^*^ ↓ NS	[143]
Osteoarthritis	H_2_S	Human serum	69 μM	45 μM NS	[143]

* statistically significant difference between control healthy and pathology; NS- non-significant difference; ^A^ it was difficult to read the exact value.

Some methods of H_2_S determination use acidification during procedure that may promote release of H_2_S from acid-labile sulfur and overestimate the concentration of free H_2_S. For this reason, it is difficult to talk about the credible and absolute value of individual RSS. However, most reports present the levels of RSS determined using the same method for control and tested biological samples, therefore, based on these data comparisons may be done. Data reporting disturbances in the RSS levels in pathological conditions refer to both low molecular weight RSS, such as H_2_S, GSSH and CysSSH as well as to the total protein persulfidation or persulfidation of specific proteins.

### Disturbances in H_2_S level

The role of H_2_S in the proper functioning of the body is well understood and disturbances in plasma H_2_S levels or its synthesis in some tissues have been described in many pathologies. In this review only a few such examples will be presented. Most authors estimated the concentrations of free H_2_S in the plasma of healthy humans within the range 15–90 µM. Rarely, the higher level of 130–150 µM was reported. Surprisingly, some authors presented that the level of free H_2_S in healthy human plasma was as low as >1 µM or less [131,144]. The reason of such a big discrepancy of the reported values is the fact that various methods were used to evaluate the amount of H_2_S. The most commonly used methods include colorimetric method based on the methylene blue formation, fluorometric assays using different fluorescence dyes (e.g. mBrB, HSip-1, 7-nitro-1,2,3-benzoxadiazole [NBD] and many others) and electrochemical determinations using ion-selective electrodes. It seems that the colorimetric method gives higher values of H_2_S compared with more specific fluorescent methods. As mentioned earlier, RSS are very difficult to assay because of their instability and due the fact that different forms of RSS coexist. Bound sulfide is released as H_2_S from its bound form (persulfides) under reducing agents or in acidic environments (acid-labile sulfur). Therefore, during the assay procedure some kind of bound sulfur can be determined as free H_2_S. Moreover, H_2_S level in plasma can be influenced by its interaction with plasma proteins or erythrocytes; therefore, the time of collection and preparation method of plasma can also influence the estimated level of H_2_S. The next factor which affects the detected H_2_S concentration is pH. In physiological pH, H_2_S exists mainly as HS^−^ and acidification of the samples facilitates the formation of H_2_S, which can be assayed by diffusion but contributes to bound sulfur release. The pH value is very important also in the determination with the ion-selective electrodes which are specific to the S^2−^ ions and, therefore, alkalization of the sample plays a crucial role. Another factor affecting the end value of the estimated H_2_S level is a good standard. Most often used standards, i.e. Na_2_S and NaHS are very susceptible to oxidation and may contain a lot of sulfane sulfur. When the standard with a relatively high sulfane sulfur content is used for S^2−^ calibration curve, the estimated values can be incorrect. Therefore, comparisons of the H_2_S and other RSS levels between healthy and pathological samples may be done only when the same method is used for control and tested samples.

A large body of evidence shows that pathogenesis of a variety of vascular diseases is connected with the down-regulation of the H_2_S pathway. In plasma of patients with hypertension as well as in patients with heart failure or ischemic heart disease, the level of H_2_S is reduced compared with the plasma of control patients [113–116]. For example, the H_2_S plasma levels of portal hypertension patients were 33.5 μM (Child–Pugh score B) and 22.2 μM (Child–Pugh score C) compared with 43.5 μM detected in control subjects (Table 2) [115]. Moreover, the plasma H_2_S level in the patients with vascular diseases was correlated with the severity of symptoms—it was significantly lower in patients with acute myocardial infarction and unstable angina compared with patients with a stable form of the disease [117]. Similarly, markedly reduced H_2_S plasma level has been reported in chronic kidney disease (CKD) patients and animal models of CKD [43,118,119]. For example, Li et al. reported the H_2_S level of 56.6 µM in healthy patients, whereas the level of H_2_S in patients with CKD was 22.9 μM and 29.1 μM in patients with or without diabetic nephropathy, respectively [120]. Additionally, several lines of evidence suggest that H_2_S can exert protective effects against CKD progression [121]. Disturbances in the synthesis and catabolism of H_2_S leading to reduced H_2_S plasma level were shown in overweight patients (22.0 μM vs. 38.9 μM in controls) and in patients with Type 2 of diabetes mellitus (DM2) (10.5 μM vs. 38.9 μM in controls) [122,123]. Moreover, it was found that low H_2_S levels in patients with DM2 correlated with a higher risk of cardiovascular disease [124]. Similar results were obtained in rat and mouse animal models, where the level of H_2_S was lower in individuals with induced diabetes, both in plasma and in some organs, such as the heart, vessels, kidneys, bone marrow and central nervous system [123,125,126]. On the other hand, studies in a model of induced diabetes in rats showed a higher concentration of H_2_S in the liver and pancreas compared with healthy control, which was accompanied by an increase in the expression of enzymes responsible for H_2_S synthesis in these organs [127,128].

Accumulating evidence demonstrated an important role of H_2_S in neurological diseases. Parkinson’s disease (PD) is manifested by motor system abnormalities, and decreased H_2_S production caused by down-regulation of CBS in the substantia nigra in PD animal models has been documented. Moreover, a decline of H_2_S level was observed in the striatum and plasma in the mouse models of PD (Table 2)[129]. Alzheimer’s disease (AD) leads to the cognitive dysfunction and loss of memory. It has been reported previously that plasma level of free H_2_S tends to be decreased in AD patients (34 μM vs. 45 μM in controls) [130], while another study published recently has not confirmed this suggestion. The latter study showed no differences in the free plasma H_2_S level in AD patients compared with the plasma of control individuals; however, disturbances in the total sulfide (acid-labile and bound) have been found by these authors in the AD patients [131]. Amyothrophic lateral sclerosis (ALS) is a neurodegenerative disease causing a selective degeneration of upper and lower motor neurons. Higher levels of H_2_S have been found in the spinal fluid of ALS patients when compared with the controls (7.53 mg/l vs. 3.64 mg/l in controls) (Table 2). Similarly, an increase in the tissue levels of H_2_S was also observed in a mouse model of ALS [132]. An elevated H_2_S production and increased urine level of thiosulfate, the main product of H_2_S catabolism, were also found in Down syndrome, a disease caused by trisomy of chromosome 21 [133–135]. The role of RSS in neurological diseases has been recently described in detail [136].

In the case of asthma and chronic obstructive pulmonary disease (COPD), deviations in the physiological level of H_2_S were also documented, however, the published data are divergent. Some studies found that serum H_2_S levels in asthmatic children were significantly decreased compared with those in non-asthmatic children (44.2 μM vs. 52.6 μM) [137], while other authors reported elevated serum H_2_S levels in severe and non-severe asthmatic patients compared with healthy subjects (280–283 μM vs. 152 μM) [138]. The diminished expression of CSE was also reported in the lung of mice in animal model of asthma [139]. There can be some possible reasons for this discrepancy including different methods used for H_2_S assay and various factors which can influence the H_2_S level, such as exacerbation of disease and degree of inflammation. In parallel, the level of H_2_S in asthmatic patients measured in the sputum was clearly elevated compared with healthy donors: 26–27 μM vs.11.4 μM [138,140] and 31.9 μM vs 19.7 μM [141,142], (Table 2). It seems that sputum H_2_S particularly correlates with the degree of airflow limitation and it has been suggested that H_2_S has potential as a novel biomarker for COPD and asthma [140]. It has also been found that mRNA and protein levels of MST were significantly reduced in cartilage of patients suffering from osteoarthritis (OA) compared with healthy donors. It was accompanied with a lower H_2_S biosynthesis in OA cartilage; however, no differences were found in the H_2_S concentration in serum from OA patients and OA-free donors (Table 2) [143].

More recently Dominic et al. studied the levels of H_2_S in plasma of COVID-19 patients and compared them with healthy controls. They found that plasma levels of free H_2_S were significantly reduced in patients suffering from COVID-19 in comparison with healthy controls (0.31 μM vs. 0.19 μM) [144]. It was true in both Caucasian and African American patients. The total sulfide levels were also decreased in the plasma of patients affected by COVID-19 (1.37 μM vs. 1.15 μM), just like plasma NO levels. The authors described also a case study of a single patient who was initially a control subject, but a few days later contracted a COVID-19 infection. The total sulfide level in this patient was significantly reduced during COVID-19 infection (from 1.11 μM before infection to 0.85 μM during infection); however following antiviral therapy with remdesivir the total sulfide level increased to 1.07 μM on the 5th day of therapy and to 1.45 μM 14 days post-treatment. The authors performed receiver-operating characteristic analysis (ROC) to determine the accuracy of H_2_S as an indicator of COVID-19. The analysis of plasma H_2_S between COVID-19 patients and controls revealed areas under the curve (AUC) of 0.87 (*P*<0.0001) for free sulfide and 0.75 (*P*<0001) for total sulfide. It suggests that especially free H_2_S can be regarded as a strong predictor of COVID-19 in an overall population; however, the AUC values were a little higher for Caucasian than for African American patients [144]. Besides free and total sulfide, the authors assayed also acid-labile sulfur, which can release free H_2_S under acidic conditions and consists mainly of iron-sulfur clusters contained in iron-sulfur proteins. The level of acid-labile sulfur was significantly decreased in patients with COVID-19 when compared with healthy controls (0.45 μM vs. 0.59). This study for the first time reported the reduction in two biochemical sulfide forms (H_2_S and acid-labile sulfur) in COVID-19 patients compared with healthy controls; however, it is only a single study and more research is needed to confirm this thesis. On the other hand, a study by Mete et al. revealed the decrease in total and native (reduced) thiols in plasma of patients with COVID-19 compared with healthy controls (282.5 μM vs. 410.5 μM for total and 169.7 μM vs. 279.0 μM for reduced thiols) [145]. This fact suggests that in the course of COVID-19 the availability of substrates for H_2_S synthesis: L-cysteine and homocysteine is limited what seems to confirm the diminished synthesis of H_2_S. Renieris et al. studied the level of H_2_S in serum of patients with COVID-19 on the 1st and 7th day after hospital admission. This study showed a correlation between the serum H_2_S level and the severity of the disease progression and final outcome. The mortality of patients with COVID-19 was significantly higher among patients with decreased H_2_S level, while in those who survived a higher serum level of H_2_S was detected on the 1st (≈195 μM for survivors vs. ≈130 μM for non-survivors) and 7th day after admission to hospital (≈190 μM for survivors vs. ≈75 μM for non-survivors) [146]. This study also revealed that serum H_2_S level was negatively correlated with the level of C-reactive protein (CRP) and IL-6 considered as the main and relevant parameter in predicting the most severe course of respiratory failure, lung injury and death in COVID-19 [147].

On the other hand, the above-cited study by Dominic et al. also performed analysis of correlation between the plasma H_2_S level and severity of COVID-19 course (estimated by cardiac injury, thrombosis and inflammatory) and found an increase in total H_2_S level in patients with severe course of COVID-19 compared with the patients with mild-to-moderately severe course of the illness [144]. These conflicting results could be due to a difference between plasma and serum, method of H_2_S estimation and other important factors affecting H_2_S level (e.g. age or accompanying diseases). It is difficult to estimate the impact of COVID-19 on plasma H_2_S level since most of patients admitted to the hospital due to SARS-CoV-2 infection suffer from other diseases associated with H_2_S disorders including DM, COPD, CKD or cardiovascular disease. In the study of Dominic et al., 75% of COVID-19 patients had hypertension while DM was recognized in 42% of patients [144]. In the Renieris study, the situation was clearer since in this group only 8% of patients suffered from DM and 21% from cardiovascular disease including chronic heart failure and coronary heart disease. Severe respiratory failure was the most frequent failure in this group of patients and affected nearly 34% of all patients [146]. The next aspect that should be taken into consideration is age of the study participants. In the study of Dominic et al., patients were in the wide age range 18–89, in the Renieris study there is an information that 47% of patients were 64 or more years old. As reported by Giustarini et al., the low molecular weight thiols and protein thiol/disulfide ratio declines in human plasma with age [148]. In the case of infection induced by SARS-CoV-2 with associated inflammation, this decline with age can be even more rapid. To clarify the correlation between plasma H_2_S levels, severity of COVID-19 and final outcome additional investigations are required.

### Disturbances in low molecular weight persulfide and polysulfide levels

Like in the case of H_2_S, disturbances in the endogenous levels of low molecular weight persulfides and polysulfides can be associated with certain diseases, although this area is much less explored. For example, it has been found that patients with diabetic retinopathy show elevated levels of CysSSH in aqueous (0.28 μM vs. 0.14 μM) and vitreous (0.18 μM vs. 0.07 μM) humor compared with healthy patients [95]. In aqueous humor, an increased level of glutathione polysulfide (GSSSG) was also observed (0.19 μM vs. 0.07 μM in controls). It seems that in the eye, some RSS are up-regulated in DM, however, these disturbances were not reflected in persulfide levels in plasma of these patients [95].

A study by Numakura et al. was designed to investigate endogenous levels of low molecular weight persulfides in the lungs in patients with chronic obstructive pulmonary disease (COPD) and to compare them with control patients. They have found reduced levels of GSSH and CysSSH in lung-resident cells and epithelial lining fluid derived from COPD patients airways (Table 2) [93]. Similarly, a decrease in the level of total (nonprotein and protein) persulfides and polysulfides was observed in the sputum of the patients suffering from COPD overlapped with asthma when compared with healthy controls (5.2 arbitrary units [AU] vs. 16.4 AU) [94]. It is interesting especially in the context of previously cited reports about the elevated H_2_S levels in the sputum of asthmatic patients. It seems that in the case of asthma and COPD disturbances in the levels of H_2_S and persulfides may result from imbalance between H_2_S and its storage in the form of persulfides rather than up- or down-regulation of RSS synthesis; however, further studies are needed to clarify this problem.

In the COVID-19 cases, there was no specific research to estimate low molecular weight persulfides or polysulfides; however, one above-mentioned paper by Dominic et al. tested also the level of bound sulfane sulfur in the plasma of COVID-19 patients. This form of reactive sulfur releases H_2_S under reducing conditions and includes various persulfides and polysulfides. This study did not reveal significant differences in the level of bound sulfane sulfur in the plasma of patients with COVID-19 when compared with the controls (Table 2) [144]. It means that infection induced by SARS-CoV-2 affects only free or total H_2_S without the effect on the bound forms of sulfur. It is also possible that other analytical methods more specific to persulfides or polysulfides could be more appropriate to demonstrate a disturbance in the level of these RSS in patients with COVID-19.

### Disturbances in protein persulfidation

Protein persulfidation is well described in the literature and disturbances in the level of protein persulfidation associated with various pathological conditions are much better known than those connected with the level of low molecular weight persulfides. This problem is particularly important as persulfidation plays a protective role and it most often affects protein activity. It makes protein persulfidation an important regulatory process, and especially in the case of specific protein targets, it can be essential in maintaining the health of the body and in treating some disease.

It has been reported that the total protein persulfidation decreases with aging. It is known that during aging an increase in reactive oxygen species (ROS) production with an accompanying decrease in antioxidant defense is observed [149]. It results in aggravation of oxidative damage and promotes development of age-related diseases, such as Parkinson’s disease and Alzheimer’s disease. Persulfides possessing powerful antioxidant properties and thiols that have weaker antioxidant properties but exist in higher concentrations in cells, are together an important cellular defense system against oxidative injury. Oxidation of cysteine -SH groups in proteins to sulfonic acids (-SO_3_H) can led to the loss of protein's activity, so protein persulfidation is regarded as a reversible modification and a kind of protection against irreversible oxidation [150]. Unfortunately, the levels of total plasma sulfane sulfur and total cysteine, the main substrate for RSS synthesis, decline with age [151]. Moreover, a study by Zivanovic et al. using rats of different ages found that protein persulfidation levels in brain extracts was reduced with age reaching approximately 50% in 24-month-old rats relative to the level of persulfidation observed in 1-month-old rats. It was accompanied by the reduced brain expression of CSE, CBS and MST. Additionally, these authors reported also an age-related decrease in protein persulfidation and expression of CSE and MST in the hearts of animals [152].

Aging is regarded as one of the main risk factors for development of neurodegenerative diseases. It has been shown that persulfidation process is dysregulated in some neurodegenerative disorders, such as PD and AD [136]. PD which is connected mainly with lesions in the motor nervous system, can be caused by a mutation of parkin protein gene. Parkin acts as a ubiquitin E3 ligase and its activity has been documented to decrease in PD [153]. Then, it has been demonstrated that parkin is persulfidated by physiological RSS and this process enhances its catalytic activity through protecting it from inactivation by S-nitrosylation. In the brains of patients with PD, a 60% decrease in parkin persulfidation was found what confirms continuing reduction of parkin activity in PD and suggests that it can be a pathological factor contributing to PD development [154].

Recently, Giovinazzo et al. studying the molecular mechanisms of neuroprotective effects of H_2_S in AD, reported that the activity of CSE, which can bind wild-type microtubule-associated protein Tau, was diminished in the 3xTg-AD mouse model as well as in AD human brains. In physiological conditions, RSS produced by CSE enhanced catalytic activity of Tau by persulfidation of its kinase, glycogen synthase kinase 3β (GSK3β) thereby preventing the hyperphosphorylation of Tau. The authors revealed that sulfhydration of GSK3β was diminished in AD by nearly 50%, while administration of RSS donors to 3xTg-AD mice, which prevent hyperphosphorylation of Tau, ameliorated motor and cognitive deficits in these mice [155].

It seems that persulfidation of specific proteins has a great significance not only in chronic neurodegenerative diseases but in many other pathologies. In the cardiovascular system, vasorelaxation is the most important activity of RSS. Among proteins involved in this process, ATP-sensitive potassium channels (*K*_ATP_) and TRPV4 channels that can activate Ca^2+^ ion-activated K^+^ (*K*_ca_) channels have been shown to be regulated by persulfidation [156]. The decreased level of H_2_S found in patients with hypertension [117,157] suggests that in this pathology the level of protein persulfidation is also decreased, especially of the aforementioned channels. The administration of RSS donors has been shown to lead to persulfidation of specific cysteine residues in the *K*_ATP_ channel, inducing hyperpolarization and vasorelaxation [158].

Kelch-like-ECH-associated protein 1 (Keap 1) is a repressor protein that binds to nuclear factor erythroid 2-related factor 2 (Nrf2) and promotes its degradation by the ubiquitin proteasome pathway. The Keap1–Nrf2 pathway is an important regulator of protective response to electrophilic and oxidative stress [159]. Keap1 is a Cys-rich protein, and its persulfidation has been well documented. It has been shown that persulfidation of Keap 1 by RSS suppressed diabetes-accelerated atherosclerosis [123] and showed protective effect against cellular aging [160]. Recently, it has also been reported that persulfidation of Keap 1 in the livers of patients with fatty liver under diabetic conditions was decreased [161].

Disturbances in protein persulfidation have been recently proposed as a mechanism that occurs in obesity. In this pathology, hypertrophy of the adipose tissue leads to oxidative stress that favors oxidation of Cys-SH residues to sulfenic acids. The latter can react with H_2_S forming protein persulfides. However, in the obesity a decreased synthesis of H_2_S means an oxidizing environment in which protein sulfenic acids are oxidized to sulfonic acid leading to protein inactivation [162]. This hypothesis seems to be confirmed by a recent study that determined protein persulfidation in various tissues of mice under dietary restriction (DR) [163]. It is well documented that DR increases lifespan of organisms [164] but Bithi et al. found that DR in mid-life could boost H_2_S production and increase protein persulfidation in many tissues. They observed enriched protein persulfidation in the liver, kidney, muscle and brain, while in the heart a decrease in protein persulfidation was observed. DR did not affect protein persulfidation in plasma of mice used in this experiment [163]. Moreover, the same authors performed a similar study introducing DR in late-life which confirmed that fasting even in late-life increased protein persulfidation in the liver, kidney, skeletal muscle and brain, although not always to the same extent as mid-life DR intervention. In this study, a decrease in protein persulfidation in the heart was also observed [165]. Interestingly, these effects were sex-dependent—they were observed only in male individuals. It suggests that the protein persulfidation is a complex process that is influenced by hormones and more studies aimed to elucidate this phenomenon is required.

## Reactive sulfur donors

As discussed above, the disturbances in homeostasis of RSS, including H_2_S, persulfides and polysulfides are involved in aging and many different pathologies. For this reason, effective and safe precursors are being developed. It is impossible here to describe all of studied compounds to be used as H_2_S releasing agents or per- and polysulfide prodrugs. It is clear that inorganic sulfides and polysulfide salts, like Na_2_S and Na_2_S_*n*_ used commonly as experimental H_2_S and polysulfides donors are not suitable for use in *in vivo* studies due to fast and uncontrollable release of RSS, what is dangerous especially in the case of free H_2_S.

### H_2_S donors

**Figure 12:**
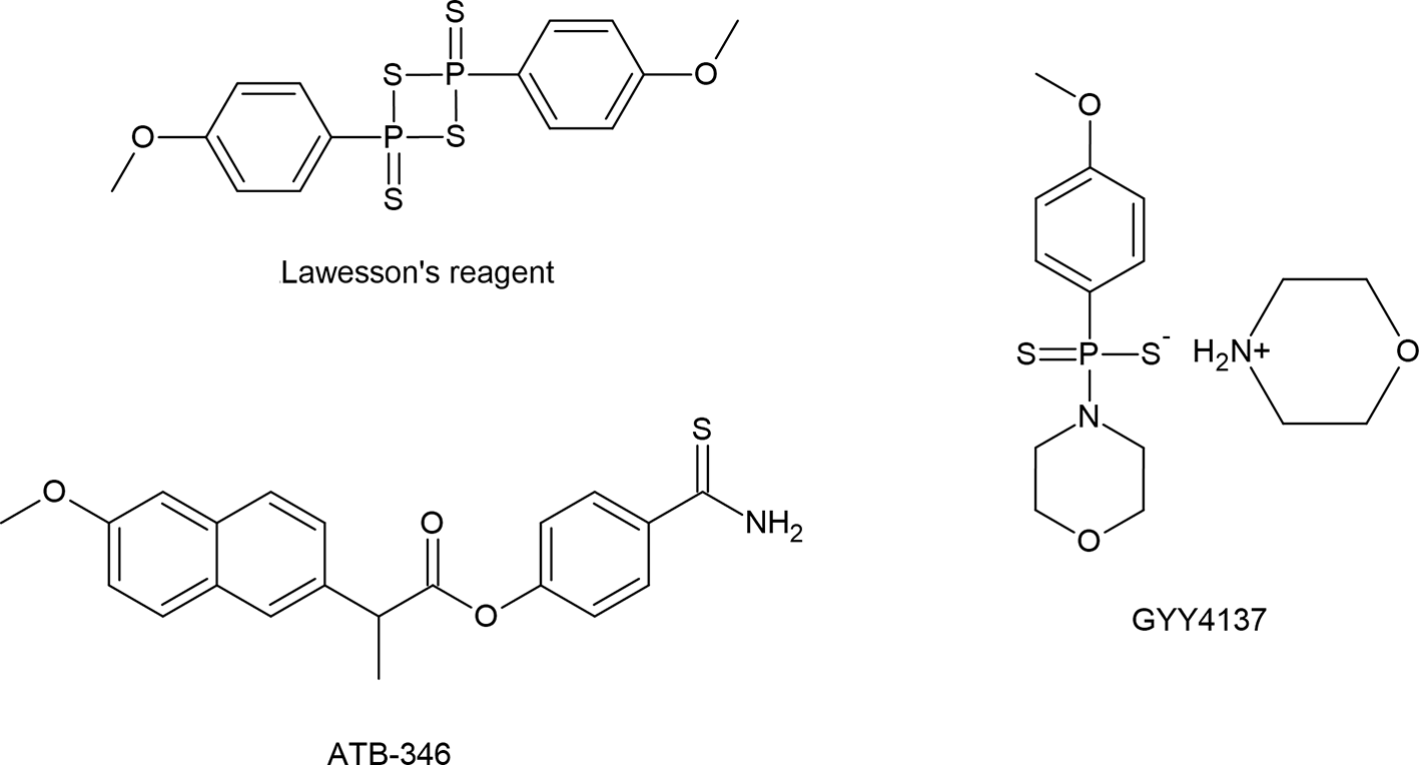
Structures of popular H_2_S donors

Among all designed donors of various sulfur species, studies aimed to develop effective donors of H_2_S were the earliest and are the most advanced. They led to development of commonly known and often used: sodium polythionate **SG-1002**, which in fact is a mixture of various sulfur compounds including elemental sulfur and acts as an RSS donor rather than as a specific H_2_S donor, **Lawesson’s reagent** (2,4-bis(4-methoxyphenyl)1,3,2,4-dithiaphosphetane-2,4-disulfide) [166,167], and a new generation, slow-releasing H_2_S donor morpholin-4-ium-4 methoxyphenyl(morpholino)phosphinodithioate known as **GYY4137** [167,168] (Figure 12). Then, many other reagents have been proposed that can release H_2_S using different mechanisms, of which the following should be mentioned here: H_2_S donors controlled by pH, hydrolysis-based donors, redox-activated H_2_S donors, photosensitive or photoactivated H_2_S donors and H_2_S donors activated by esterase. Moreover, donors based on labile S-N bonds have been developed, and it was revealed that thiols (e.g. Cys) are essential to trigger H_2_S release from these molecules [169]. The next important group of H_2_S prodrugs, some of which entered the clinical phase, includes H_2_S-releasing modifications of many clinically used drugs with the well-known **ATB-346** which is a H_2_S-releasing derivative of naproxen [170,171] (Figure 12). An important representative of H_2_S precursors carbonyl sulfide (COS) is hydrolyzed in mammalian cells to H_2_S by carbonic anhydrase [172]. COS production in mammalian cells has yet to be confirmed; however, COS application as H_2_S donor for biological applications can be accepted due to enzymatic conversion of COS to H_2_S [173]. The list of compounds designed and tested as potential H_2_S donors is much longer and it was the subject of several review papers [174–178].

Recently, it has been suggested that the mechanism of action of the well-known drug N-acetylcysteine (NAC) is connected with its conversion to H_2_S and other RSS containg sulfane sulfur [179]. NAC easily penetrates into the cells where it is deacetylated to Cys, the main substrate for H_2_S synthesis. Ezerina et al. were the first who noticed that NAC triggered the production of intracellular H_2_S and other sulfane sulfur-containing compounds [180]. NAC is clinically used in various pathologies including neurological disorders, liver failures, spermatogenesis disorders and the spectrum of its pharmacological activity is constantly expanding [181–183] but the mechanism of its action is not fully explained.

A few clinical studies reported the effectiveness of NAC as an adjunctive therapy in COVID-19. Some patients with moderate or severe COVID-19 pneumonia received orally NAC apart from standard care. The obtained results revealed that NAC slowed down the progression to severe respiratory failure, reduced inflammation and caused a clinical improvement when compared with the control patients who received only standard treatment without NAC. A lower mortality was also observed in the group of patients treated with NAC [184,185]. Unfortunately, none of these reports investigated the plasma H_2_S level in patients with COVID-19 before and during therapy of NAC, so it could be only hypothesized that beneficial action of NAC in patients with severe COVID-19, is largely due to the increase in H_2_S concentration.

### Persulfide donors

One of the most important mechanism of RSS action in biological systems is related to persulfidation of proteins. This reaction can be triggered by H_2_S if target -SH groups in proteins are reversibly oxidized to sulfenic acid or S-nitrosothiols. When the protein -SH groups are in reduced state, only persulfides or polysulfides can sulfhydrate target proteins. It is assumed that persulfides and polysulfides are regarded as more efficient persulfidating agents than H_2_S. As mentioned earlier, persulfides are also more active and safer sulfur species than H_2_S what makes them good candidates for sulfur redox signaling. However, due to their low stability it is not easy to use them directly for biological application. Therefore, some efforts have been made in making persulfide donors suitable for use in biological systems.

**Figure 13:**
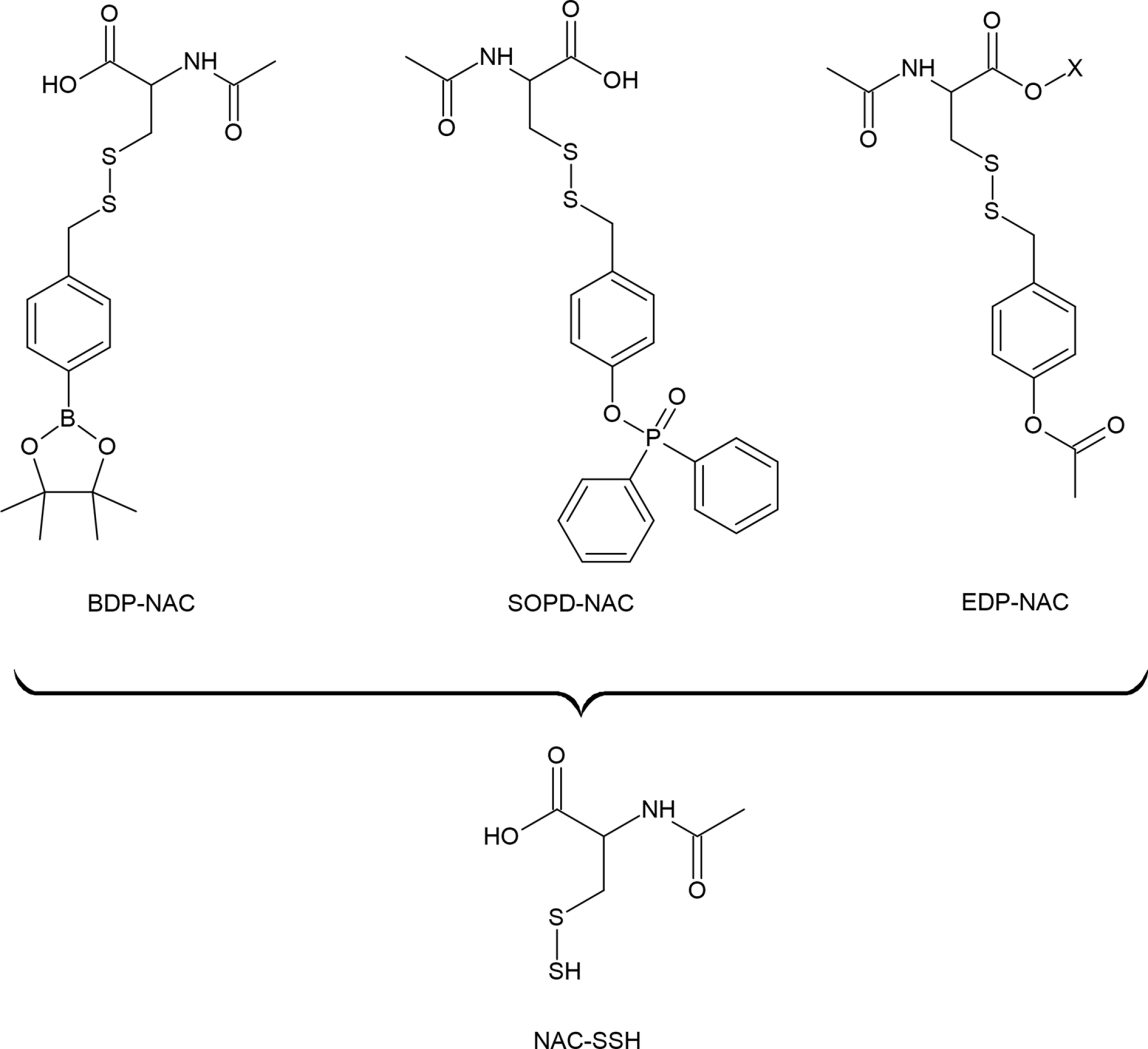
Examples of various persulfide prodrugs

Currently, several small molecules have been described as persulfide donors, which release persulfides in response to a different stimuli. A series of esterase-sensitive persulfide prodrugs was developed including a GSSH donor [186]. Powell et al. developed and reported the ROS-responsive persulfide donor termed Bpin-disulfide prodrug (**BDP-NAC**) which is a derivative of NAC (Figure 13). This compounds released NAC-SSH in response to H_2_O_2_ [175]. The same research group developed then another derivative of NAC, termed superoxide-responsive, persulfide donor, **SOPD-NAC**, which reacts with superoxide radical anion (O_2_^●−^) releasing NAC-SSH (Figure 13) [187]. Continuing their research on effective persulfide prodrugs, these authors next synthesized a small molecule which acts as an NAC-SSH donor in response to esterase and termed it ester disulfide prodrug, **EDP-NAC.** Moreover, they designed and synthesized its polymeric form termed **poly(EDP-NAC)** (Figure 13). The performed experiments confirmed that polymeric form of this prodrug released persulfides much more slowly and protected cardiomiocytes more effectively than EDP-NAC [188]. More examples of persulfide prodrugs including photosensitive agents, molecules activated by ROS and other structures were described in the literature [68,178,186,189–193].

### Polysulfide donors

In relation to the above-described compounds, NAC-derivatives have been studied as persulfide prodrugs, and NAC polysulfides were tested as polysulfide prodrugs. It was demonstrated that NAC polysulfides (Figure 14) efficiently increased intracellular levels of RSS, however, a significant increase in the content of persulfides, such as CysSSH and GSSH was also observed [194]. It means that NAC polysulfides are good, stable and effective RSS precursors but they are not selective for polysulfides. It seems to be a general rule for per- and polysulfide prodrugs due to a very close relationship between these two kinds of RSS. Moreover, the equilibrium between H_2_S, persulfides and polysulfides is dynamic and depends on various factors including pH and redox status of the cells. It was also confirmed by a study utilizing a synthetic slow-releasing RSS donor, which is chemically derived from penicillamine [191]. This compound generates a persulfide and a mixture of polysulfides as end-products. Moreover, it can also release H_2_S, but only in the presence of thiols, such as Cys-SH or GSH [195].

**Figue 14:**
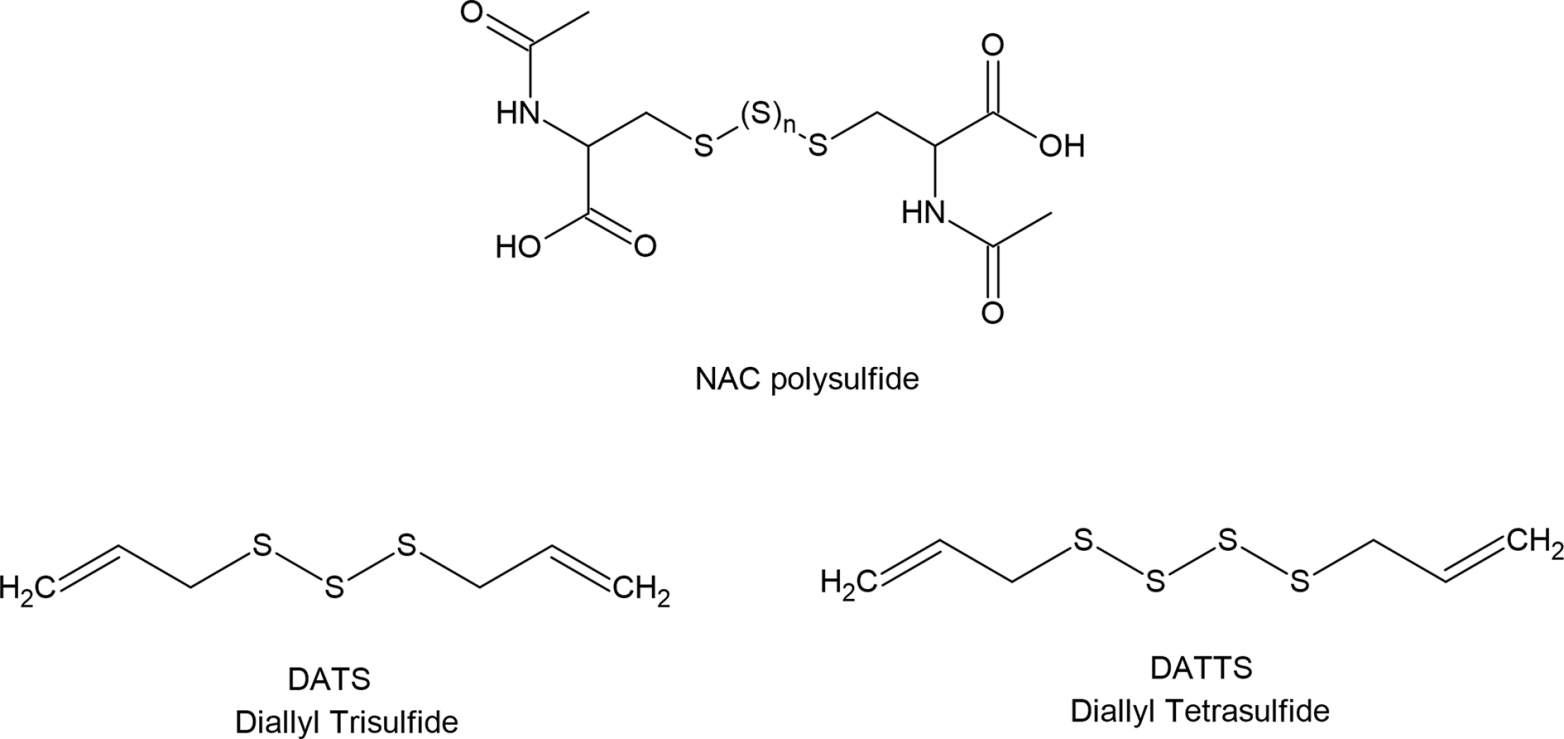
Examples of polysulfide donors

Among natural rich sources of polysulfides, garlic deserves special attention, as it contains organopolysulfides, including diallyl trisulfide (DATS) and diallyl tetrasulfide (DATTS) which can be used as RSS donors (Figure 14). These compounds after polysulfide bond cleavage form persulfides (RSS_n_H), which can further react with thiols leading to H_2_S production [196]. This reaction can be repeated and other polysulfides are produced as byproducts. The effectiveness of DATS as an RSS precursor was documented *in vivo* in animal models. DATS administered intraperitoneally to mice elevated the total sulfane sulfur pool (persulfides and polysulfides) and increased the activity of enzymes involved in their metabolism in the liver and kidney [197,198]. Interestingly, Tocmo et al. studied the effect of cooking on garlic's organopolysulfides and H_2_S-releasing activity. They found that the amounts of allyl polysulfides increased in crushed garlic boiled for 6–10 min; however, longer thermal treatment decreased their concentrations [199]. Garlic-derived allyl polysulfides as well as isothiocyanates derived from Brassicae have been recognized as polysulfide or H_2_S donors of great pharmacological potential. Recently, growing attention has been focused on these dietary organosulfur compounds in relation to the possibilities of use in the treatment of diseases with reduced RSS synthesis [200,201]. Moreover, garlic-derived polysulfides are proposed as a potential therapeutic food to fight COVID-19 [202–204].

### H_2_S_*n*_ donors

**Figure 15:**
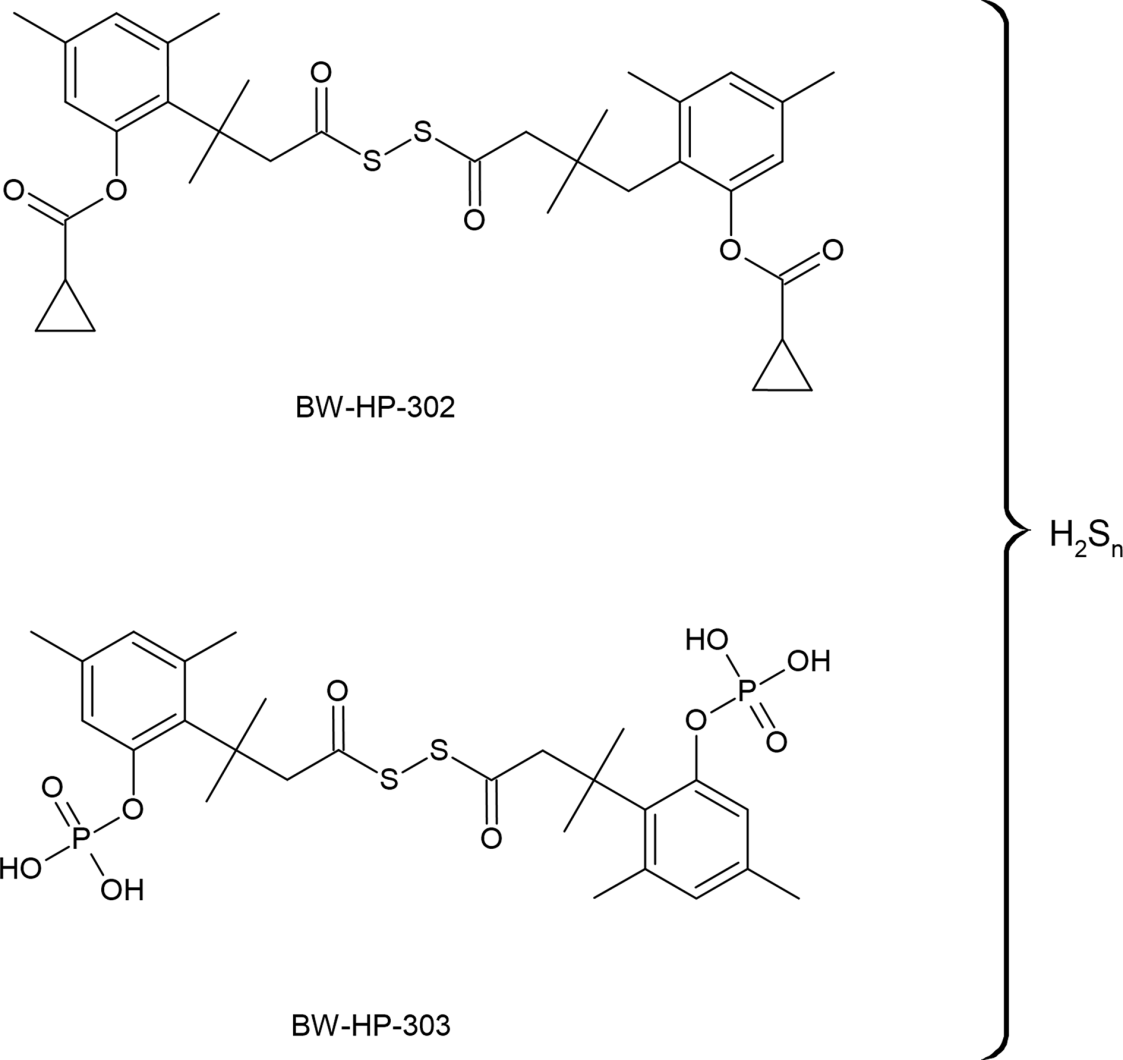
Structures of the two developed H_2_S_*n*_ donors

Like in the case of H_2_S, persulfides and polysulfides, slow and controllable donors of inorganic polysulfides (H_2_S_*n*_) are looked for but this area is not well explored. Yu et al. developed esterase- and phosphatase-sensitive H_2_S_2_ prodrugs with tenable release rates, termed **BW-HP-302** and **BW-HP-303**, respectively (Figure 15). The utility of these compounds in RSS donating was confirmed by S-persulfidation of glyceraldehyde 3-phosphate dehydrogenase (GAPDH) [205]. H_2_S_*n*_ donors, like BW-HP compounds, are based on the template of highly reactive acyl disulfide, they can form polysulfides and release H_2_S under the influence of thiols [206]. Acyl disulfides can also react with nucleophiles, like amines, which suggests that these H_2_S_*n*_ donors in biological systems may react with naturally existing molecules, like amino acids [102]. Recently, Xu et al. demonstrated that diacyl disulfides could produce H_2_S_2_ in reaction with various nucleophiles including amines in both aqueous and organic environment [206].

The more is known about the biological role of RSS and disorders of homeostasis in many pathological conditions, the greater the need to develop slow- and controlled-release RSS donors which could be used in experimental studies and, in the longer term, could be applicable in clinical trials to treat some pathologies. The list of potential developed H_2_S donors is impressive, but only a few of them in experimental studies showed features predisposing them to clinical applications. Among the recently developed precursors, NAC derivatives seem to be the most promising molecules. As NAC is a drug approved for clinical use, it is quite safe and well tolerated. Therefore, stable NAC derivatives that are easily transformed in cells into NAC persulfides, providing RSS for protein persulfidation and a powerful antioxidant and reductive force, open up possibilities for their use to treat diseases. The second promising source of RSS derives from polysulfides and isothiocyanates naturally occurring in garlic and Brassicae. A diet rich in these foods may be effective in protecting against diseases associated with reduced RSS synthesis, such as cardiovascular diseases, cancer or aging.

## Reactive sulfur inhibitors

For understanding the biological function of H_2_S and other forms of RSS, pharmacological inhibitors of the main enzymes involved in synthesis of H_2_S and other RSS may play a helpful role. They include three sulfurases: two PLP-dependent enzymes: CSE and CBS as well as one PLP-independent MST. Moreover, some reports mentioned above and cited in Table 2 have documented that activity of these enzymes is up-regulated in some pathologies leading to overproduction of H_2_S and other RSS. Therefore, compounds able to selectively inhibit individual enzyme will be needed.

In the case of CSE, which is a major source of H_2_S in peripheral tissues and cardiovascular system, the most popular compounds used to inhibit this enzyme include DL-propargyl-glycine (PAG), β-cyano-L-alanine (BCA) and L-aminoethoxyvinylglycine (AVG) [174]. These compounds preferentially inhibit CSE, however, they have been shown to inhibit also other PLP-dependent enzymes [207]. Of these compounds, PAG is the most commonly used drug of choice to pharmacologically inhibit CSE. In animal models, this compound elevated blood pressure [116] and increased: cardiac damage [208], ischemia/reperfusion injury [209] and mortality in sepsis [210]. Interestingly, Peng et al. suggested that inhibition of CSE by PAG might be a useful therapeutic intervention to prevent carotid body- driven sleep apnea [211]. Due to poor selectivity and poor efficiency (high concentration and limited ability to permeate the cell membrane) of PAG, new potent and selective inhibitors are searched for. In this aspect, Hu et al. studied recently several potential compounds and found that compound named NSC4056, which is a derivative of salicylic acid, was the most potent, active and selective CSE inhibitor [212]. This result offers new opportunities for development of new pharmacologically active remedies to selectively inhibit CSE; however, more studies *in vivo* using NSC4056 are needed.

CBS is predominantly present in the central nervous system but it has also been found in other tissues including the liver, pancreas and kidney. Despite many studies aimed to discover new, selective CBS inhibitors, to date aminooxyacetic acid (AOAA) CBS remains the most commonly used inhibitor of CBS [17]. This compound has been shown to normalize H_2_S overproduction mediated by CBS, to restore normal mitochondrial function in Down syndrome [213] and to inhibit proliferation, angiogenesis and cell bioenergetics in cancer cells [214,215]. Moreover it has been also found that AOAA reduced vascular relaxation induced by acetylcholine what confirms implication of H_2_S in the regulation of vascular tone [216]. As mentioned above, AOAA is regarded as ‘classical CBS inhibitor’; however, it has been well documented that AOAA reacts with PLP and in this way it can also inhibit other PLP-dependent enzymes including CSE [217]. Moreover, study by Asimakopoulou et al. using recombinant CBS and CSE revealed that AOAA exhibited more potency toward CSE when compared with CBS (IC 1.1 vs. 8.5 µM, respectively) [207].

Thus, AOAA and PAG, commonly used inhibitors of CBS and CSE, despite of their usefulness have the limitations associated mainly with the lack of the selectivity and low cell uptake.

For many years, pharmacological inhibitors have not been identified. L-aspartate has been documented to inhibit CAT [217], so the use L-aspartate can suppress H_2_S production by limitation of 3-MP, the substrate for MST. Recently, the role of MST/H_2_S has been studied in some aspects using the novel pharmacological, potent and selective MST inhibitor (2-[(4-hydroxy-6-methylpyrimidin-2-yl)sulfanyl]-1-(naphthalen-1-yl)ethan-1-one (HMPSNE). This compound in a concentration-dependent way suppressed H_2_S production and exerted antiproliferative effects in murine colon cancer [218]. Moreover, HMPSNE produced a bell-shaped effect on several cellular bioenergetic parameters related to oxidative phosphorylation, while other bioenergetic parameters were either unaffected or inhibited at the highest concentration of the inhibitor [218]. On the other hand, Casili et al. using HMPSNE revealed that inhibition of MST significantly enhanced lipid accumulation into the maturing adipocytes [219]. All the studied bioenergetic parameters, including ATP production, were significantly lower in mice adipocyte tissue pretreated with HMPSNE compared with untreated tissue [219]. These results are interesting and it seems that HMPSNE is a promising candidate for selective inhibition of MST; however, more studies including *in vivo* experiments are needed to confirm its effectiveness and safety.

Overproduction of RSS is found in a smaller number of diseases when compared with the deficiency of RSS synthesis. However, it seems that finding safe and selective inhibitors of enzymes participating in the RSS synthesis, in the future would enable their use in diseases, the course of which is associated with overproduction of RSS, such as Down syndrome or ALS.

## Conclusions

Mounting evidence suggest that RSS play an important role in redox regulation and are potent physiological mediators. Initially, this role was mainly assigned to H_2_S, but now it is known that these are persulfides and polysulfides, closely related to H_2_S, that are responsible for many biological effects, including protein persulfidation, and they possess more potent antioxidant and reductive properties. Since all RSS coexist in biological systems and per- and polysulfides are rather unstable molecules, research on the role of particular forms of RSS is challenging. The high reactivity of these compounds makes this task even more difficult; however, constantly developing methods and tools for specific detection of individual RSS forms offer hope for a more reliable analysis and a better understanding of the physiological role of RSS. Over the past 20 years, tremendous progress has been made in developing methods and clarifying the role of RSS in many pathologies. Moreover, many stable and efficient-release donors of RSS have been developed, however, most of them have been tested under *in vitro* conditions. Among the recently developed RSS precursors, NAC derivatives seem to be the most promising molecules for clinical use since NAC is quite safe, cheap, well-tolerated and most importantly it has been approved for clinical use. In several studies, NAC was also used to treat patients with severe COVID-19 with a success. It seems reasonable that in the aspect of SARS-CoV-2, the beneficial effect of NAC is at least partly related to RSS generation and modulation of their physiological level. This, together with documented anti-inflammatory and antiviral properties of NAC, provides the basis for considering it or its derivatives as a beneficial adjuvant therapy in the course of COVID-19 and other pathologies.

## Abbreviations

AOAA: aminooxyacetic acid
AVG: L-aminoethoxyvinylglycine
BCA: β-cyano-L-alanine
CARS: cysteinyl-tRNA synthetase
CBS: cystathionine β-synthase
CSE: cystathionine γ-lyase
HMPSNE: 2-[(4-hydroxy-6-methylpyrimidin-2-yl)sulfanyl]-1-(naphthalen-1-yl)ethan-1-one
MST: 3-mercaptopyruvate sulfurtransferase
NAC: N-acetylocysteine
PAG: DL-propargyl-glycine
PLP: pyridoxal phosphate
RSS: reactive sulfur species
